# Integrated sensory, chemical, metabolomic, and antioxidant profiling reveals coordinated quality transformation of Lapsang Souchong black tea during decade-long storage

**DOI:** 10.3389/fpls.2026.1934917

**Published:** 2026-08-20

**Authors:** Shulin Zheng, Aoge Gong, Lirong Zuo, Xueni Xie, Liting Ou, Shuangxu Yang, Xi Cheng, Xiaoyong Wang, Bo Zhang, Yutao Shi

**Affiliations:** 1 College of Tea and Food Sciences, Wuyi University, Wuyishan, China; 2 Tea Engineering Research Center of Fujian Higher Education, Wuyi University, Wuyishan, China; 3 Department of Management, Minbei Vocational and Technical College, Yanping, China; 4 College of Mechanical and Electrical Engineering, Huangshan University, Huangshan, China

**Keywords:** Lapsang Souchong black tea, long-term storage, multimodal integration, PTIO· radical scavenging, sensory quality, tea aging, theabrownins, widely targeted metabolomics

## Abstract

Long-term storage of Lapsang Souchong (LS) black tea is traditionally believed to improve its aged flavor and functional quality, but the multi-level changes underlying this process remain insufficiently characterized. In this study, LS black tea samples from eight production years (2013–2023, corresponding to 10 to 0 years of natural storage) were investigated using sensory evaluation, chemical composition analysis, antioxidant activity assays, and multimodal data integration. Electronic tongue profiling and widely targeted UPLC–MS/MS metabolomics were further performed on five representative production years to characterize instrumental taste responses and non-volatile metabolite remodeling. Quantitative descriptive analysis revealed storage-responsive taste changes, with Sour and Smooth increasing during storage and Brisk, Umami, and Sweet decreasing. Among these attributes, Sour showed the strongest monotonic association with storage duration (Spearman r = +0.994). Chemical analysis indicated storage-related tea pigment transformation, especially higher theabrownin levels and lower TR/TB ratios in long-stored samples. Metabolomic profiling identified 183 storage-associated metabolites, revealing prominent changes in lipid-derived compounds, flavonoid- and tannin-related metabolites, and pine-smoking-derived diterpenoids. Notably, abietic acid and dehydroabietic acid were markedly depleted during storage, suggesting that pinewood-smoking-related diterpenoids may serve as characteristic molecular indicators of LS storage transformation. Among the four antioxidant assays, PTIO· scavenging showed a significant negative association with storage duration (Spearman r = −0.810, *p* = 0.015), suggesting its potential relevance to the depletion or transformation of nitrogen-containing bioactive compounds. Multimodal integration using a single-latent-variable partial least squares regression model captured the major storage-related variation based on 19 predictors, with cross-validated performance of *Q^2^* = 0.864 and RMSECV = 1.370 years. Twelve predictors showed VIP values greater than 1.0 across sensory, electronic tongue, chemical, metabolomic, and antioxidant modalities. In addition, a simplified composite storage score showed strong concordance with actual storage duration across the eight production years (Spearman r = +0.976, *p* < 0.001). Overall, these findings indicate that LS black tea storage involves coordinated sensory, chemical, molecular, and functional transformations rather than simple quality deterioration, providing a quantitative basis for storage-related quality evaluation.

## 1 Introduction

Tea, prepared from the young leaves and buds of *Camellia sinensis* (L.) O. Kuntze, is one of the most widely consumed non-alcoholic beverages worldwide (Cabrera et al., 2006; Khan and Mukhtar, 2018). According to processing methods and the degree of enzymatic oxidation, tea is generally classified into green, yellow, white, oolong, black, and dark teas (Tang et al., 2019). Among these categories, black tea is one of the most important tea types in global consumption and trade. Its characteristic reddish-brown liquor color, mellow taste, and distinctive aroma are mainly formed during fermentation, in which catechins are enzymatically oxidized and polymerized into theaflavins (TF), thearubigins (TR), and theabrownins (TB) (Robertson, 1992; Tanaka et al., 2009). These pigment-related compounds, together with residual catechins, amino acids, caffeine, soluble sugars, and aroma compounds, constitute the biochemical basis of black tea quality and contribute to its sensory and functional properties (Sharma and Rao, 2009; Bag et al., 2022).

Lapsang Souchong (LS), known in Chinese as Zheng Shan Xiao Zhong, is a representative traditional Chinese black tea originating from Tongmu Village in the Wuyi Mountains of northern Fujian Province, China. It is widely recognized for its historical importance in black tea development and for its distinctive pine-smoked flavor profile (Yao et al., 2005; Su et al., 2025). During traditional processing, tea leaves are exposed to smoke generated from pine wood during withering and drying, producing characteristic smoky, pine-like, and sweet aroma notes. Previous studies have identified smoke-related volatile compounds such as guaiacol, 4-methylguaiacol, 4-ethylguaiacol, longifolene, and α-terpineol as important contributors to the sensory identity of LS (Yao et al., 2005; Su et al., 2025). Owing to its unique geographical origin, processing technique, and sensory characteristics, LS has become a specialty black tea with considerable cultural and economic value.

Long-term storage is an important factor affecting tea quality. In traditional Chinese tea culture, certain teas are believed to develop more desirable flavor and functional attributes during storage, a concept commonly associated with aged tea products (Zhang et al., 2011; Ge et al., 2021). Scientific studies have shown that tea storage is not merely a passive preservation process, but involves continuous biochemical transformations, including catechin oxidation, tea pigment conversion, lipid degradation, amino acid transformation, and changes in aroma-related compounds (Zhang et al., 2019; Wang et al., 2022). These storage-related changes have been investigated in several tea types, including Pu-erh tea, white tea, oolong tea, Qingzhuan tea, and other aged teas (Cheng et al., 2021; Hong et al., 2021; Zhao et al., 2022; Chen et al., 2025). However, the long-term storage behavior of fully fermented black teas, especially LS, remains less well understood.

Several knowledge gaps remain. First, most studies on tea aging have focused on post-fermented or lightly fermented teas, whereas systematic investigations of long-term storage in LS black tea are still limited. A recent decade-scale metabolomic comparison involving LS provided valuable information on storage-related chemical changes, but sensory, instrumental taste, chemical, metabolomic, and functional data have rarely been integrated within the same analytical framework (Pang et al., 2024). Second, existing studies often emphasize a single dimension, such as chemical composition, metabolomics, sensory quality, or antioxidant activity. This makes it difficult to determine whether storage-related quality changes in LS reflect independent degradation events or a coordinated multi-level quality transformation. Third, although individual storage-associated indicators have been reported for different teas, an interpretable quantitative framework for evaluating LS storage-related quality changes remains lacking. Such a framework would be useful for understanding tea aging biochemistry and for supporting quality evaluation, grading, storage management, and authenticity assessment.

To address these gaps, we conducted a decade-long, multi-modal investigation of LS black tea across eight production years, including 2013, 2016, 2018, 2019, 2020, 2021, 2022, and 2023, corresponding to 10, 7, 5, 4, 3, 2, 1, and 0 years of natural storage, respectively. Five complementary analytical modalities were integrated to characterize storage-related changes from sensory, chemical, molecular, and functional perspectives. Sensory changes were evaluated using traditional Chinese sensory assessment, quantitative descriptive analysis, and electronic tongue profiling. Chemical changes were assessed by measuring major quality-related components, including polyphenols, amino acids, caffeine, soluble sugars, and the TF–TR–TB pigment system. Widely targeted UPLC–MS/MS metabolomics was applied to five representative production years to characterize non-volatile metabolite remodeling, while antioxidant activity was evaluated using DPPH·, ·OH, ${{\text{O}}_{2}}^{-}\cdot$, and PTIO· scavenging assays. These datasets were further integrated using multivariate statistics, correlation analysis, PLSR modeling, and a composite storage score. Through this framework, this study aimed to characterize storage-associated quality transformation in naturally stored Lapsang Souchong black tea across a decade-long production-year series, identify storage-associated indicators including pinewood-smoking-related molecular features, and develop an interpretable quantitative basis for storage-related quality evaluation.

## 2 Materials and methods

### 2.1 Tea samples

Lapsang Souchong (LS) black tea samples from eight production years (2013, 2016, 2018, 2019, 2020, 2021, 2022, and 2023) were obtained from Wuyishan Hefeng Jiaye Tea Factory, Wuyishan, Fujian, China. All samples were produced from fresh leaves harvested from the same tea garden in Tongmu Village, Xingcun Town, Wuyishan City, Fujian Province. The raw material was Wuyi Cai Cha, a local landrace population of *Camellia sinensis* (L.) O. Kuntze var. *sinensis* native to the Wuyi Mountain region. Fresh leaves were plucked in May of each production year according to the standard of one bud with two or three leaves. All samples were processed using the traditional LS manufacturing procedure according to DB35/T 1228–2015, including withering, rolling, fermentation, drying, and pinewood smoking. Briefly, fresh leaves were sun-withered for approximately 6 h, further withered over pinewood fire at approximately 40 °C for 2.5 h, rolled at approximately 25 °C, fermented under warm and humid conditions for 8 h, and finally smoke-dried with pinewood smoke at approximately 90 °C for 12 h (Yao et al., 2005; Li et al., 2024; Su et al., 2025). After processing, the tea samples were sealed in aluminum foil bags and stored in a dry, well-ventilated warehouse protected from direct light. The warehouse temperature and relative humidity were maintained within the general ranges of ≤30 °C and ≤50%, respectively. For each production year, three independent production batches were collected as biological replicates (n = 3). All samples were retrieved in July 2023 and designated by their production years, corresponding to natural storage durations of 10, 7, 5, 4, 3, 2, 1, and 0 years, respectively. The 2023 sample was used as the freshly processed reference sample. After retrieval, all samples were transported to the laboratory and stored at −20 °C until analysis.

### 2.2 Sensory evaluation

Sensory evaluation was performed according to the Chinese National Standard for Sensory Evaluation of Tea (GB/T 23776–2018) and standard quantitative descriptive analysis (QDA) procedures (Stone et al., 2004; Liu et al., 2025).

#### 2.2.1 Traditional Chinese sensory evaluation

Traditional sensory evaluation was conducted by 15 trained tea sensory evaluators, including seven males and eight females aged 25–60 years, each with more than five years of experience in Chinese tea sensory assessment. Tea samples (3.00 g) were infused with 150 mL of freshly boiled distilled water in standardized porcelain cups for 5 min and then transferred to evaluation bowls for assessment. Sensory evaluation was performed in a dedicated sensory room maintained at 20–22 °C and 60–70% relative humidity under standardized illumination. Five sensory dimensions, including appearance, liquor color, aroma, taste, and infused leaves, were evaluated using a 100-point scale. The overall sensory score was calculated as a weighted score according to GB/T 23776–2018.

#### 2.2.2 Quantitative descriptive analysis

QDA was used to further characterize the taste profile of the tea infusions. Nine taste attributes were evaluated: Strong, Thick, Umami, Brisk, Sweet, Bitter, Astringent, Smooth, and Sour. Attribute definitions and reference standards were established during three training sessions before formal evaluation. Each attribute was rated on a 0–7 continuous intensity scale, where 0 indicated “not perceptible”, 4 indicated moderate intensity, and 7 indicated “very strong”. All samples were evaluated by the same fifteen panelists in triplicate on separate days using a randomized and blinded design. Samples were coded with three-digit random numbers, and warm distilled water and unsalted crackers were provided between samples for palate cleansing. The final QDA score for each attribute was calculated as the mean value across panelists and replicates.

### 2.3 Chemical composition analysis

Nine major taste-related chemical components, including water extract, tea polyphenols, free amino acids, caffeine, soluble sugars, total flavonoids, theaflavins (TF), thearubigins (TR), and theabrownins (TB), were determined. All analyses were performed using three biological replicates, and results were expressed as percentage (%) of dry weight unless otherwise stated. Absorbance measurements were conducted using a UV–visible spectrophotometer (UV-1800PC, Meipuda, Shanghai, China).

#### 2.3.1 Water extract content

Water extract content was determined according to GB/T 8305–2013. Briefly, 2.00 g of ground tea sample was extracted with boiling distilled water, filtered, and the residue was dried at 120 °C to constant weight. Water extract content was calculated gravimetrically.

#### 2.3.2 Tea polyphenols

Total tea polyphenols were determined using the Folin–Ciocalteu colorimetric method according to GB/T 8313–2018. Ground tea samples were extracted with 70% aqueous methanol at 70 °C, and the extract was reacted with Folin–Ciocalteu reagent and sodium carbonate solution. After incubation in the dark, absorbance was measured at 765 nm. Gallic acid was used as the calibration standard.

#### 2.3.3 Free amino acids

Total free amino acids were determined using the ninhydrin colorimetric method according to GB/T 8314–2013. Tea extract was reacted with ninhydrin solution and phosphate buffer at 100 °C, cooled, and diluted. Absorbance was measured at 570 nm, and L-theanine was used as the calibration standard.

#### 2.3.4 Caffeine

Caffeine content was determined using the ultraviolet spectrophotometric method according to GB/T 8312–2013. Tea extract was purified with magnesium oxide and diluted with distilled water. Absorbance was measured at 274 nm, and caffeine was used as the calibration standard.

#### 2.3.5 Soluble sugars

Total soluble sugars were determined using the anthrone–sulfuric acid colorimetric method with minor modifications (Morris, 1948; Yemm and Willis, 1954; Li et al., 2017). Briefly, tea extract was reacted with freshly prepared anthrone reagent in concentrated sulfuric acid, heated in a boiling water bath, cooled to room temperature, and measured at 620 nm. Glucose was used as the calibration standard, and results were expressed as glucose equivalents.

#### 2.3.6 Total flavonoids

Total flavonoids were determined using an aluminum chloride colorimetric method with minor modifications (Chang et al., 2002; Magalhães et al., 2012). Briefly, tea extract was mixed with AlCl_3_ solution, and the absorbance of the flavonoid–Al (III) complex was measured at 420 nm. Rutin was used as the calibration standard, and results were expressed as rutin equivalents.

#### 2.3.7 Theaflavins, thearubigins, and theabrownins

Theaflavins, thearubigins, and theabrownins were determined using the systematic analysis method originally developed by Roberts and Smith (1961) and further refined by Robertson (1992). Briefly, ground tea samples were extracted with boiling distilled water and filtered. The filtrate was sequentially partitioned with ethyl acetate and n-butanol to obtain different pigment fractions. Absorbance values were measured at 380 nm and 460 nm, and TF, TR, and TB contents were calculated according to Robertson’s formulae. The TR/TB ratio was subsequently calculated to characterize the relative transformation of red and brown tea pigments during storage.

### 2.4 Electronic tongue analysis

Electronic tongue analysis was conducted using a TS-5000Z taste sensing system (Insent Inc., Atsugi, Kanagawa, Japan) equipped with seven lipid membrane sensors and two reference electrodes, following previously reported methods with minor modifications (Chen et al., 2008; He et al., 2009). The recorded taste-related responses included Sourness, Bitterness, Astringency, Umami, Saltiness, Sweetness, Aftertaste-A, Aftertaste-B, and Richness. Tea infusions were prepared by brewing 3.00 g of ground tea sample with 150 mL of boiling distilled water for 5 min. After cooling to room temperature, the infusion was filtered through Whatman No. 1 filter paper and diluted 1:1 with distilled water. Approximately 70 mL of the diluted infusion was used for measurement. Each sample was measured in triplicate at 25 ± 1 °C. Sensors were cleaned with reference solution between measurements, and system stability was verified using reference solutions before and after each sample batch. Mean sensor responses were used for PCA and correlation analyses.

### 2.5 Widely targeted metabolomic analysis

Widely targeted metabolomic analysis was performed on five representative production years, including 2013, 2016, 2019, 2022, and 2023, with three biological replicates per year. The analysis was conducted by Metware Biotechnology Co., Ltd. (Wuhan, China) following previously described procedures with minor modifications (Chen et al., 2013; Zhou et al., 2022).

#### 2.5.1 Sample preparation

Freeze-dried tea samples were ground into fine powder using a mixer mill (MM 400, Retsch GmbH, Haan, Germany) with zirconia beads. Approximately 100 mg of powder was extracted with 1.2 mL of 70% aqueous methanol. The extract was vortexed, centrifuged at 12,000 rpm for 3 min at 4 °C, and filtered through a 0.22 μm membrane before UPLC–MS/MS analysis.

#### 2.5.2 UPLC–MS/MS conditions

Metabolomic profiling was performed using an ExionLC AD UPLC system coupled to a QTRAP 6500+ mass spectrometer (AB Sciex, Framingham, MA, USA). Chromatographic separation was carried out on an Agilent SB-C18 column (1.8 μm, 2.1 × 100 mm; Agilent Technologies, Palo Alto, CA, USA) at 40 °C. The mobile phases consisted of water containing 0.1% formic acid as solvent A and acetonitrile containing 0.1% formic acid as solvent B. The gradient program was as follows: 5–95% B from 0 to 9 min, 95% B from 9 to 10 min, 95–5% B from 10.0 to 11.1 min, and 5% B from 11.1 to 14.0 min. The flow rate was 0.35 mL/min, and the injection volume was 4 μL. Mass spectrometry was performed in both positive and negative electrospray ionization modes. Source parameters were set as follows: source temperature, 550 °C; ion spray voltage, +5,500 V in positive mode and −4,500 V in negative mode; curtain gas, 35 psi; and collision gas, medium. Multiple reaction monitoring mode was used for metabolite quantification, with declustering potential and collision energy optimized for each transition.

#### 2.5.3 Metabolite identification and data processing

Metabolites were identified by matching precursor ions, product ions, and retention times against the Metware Database (MWDB), a self-constructed metabolite database containing authentic standards and reference compounds (Chen et al., 2013). Peak integration and quantification were performed using MultiQuant 3.0.3 software (AB Sciex), and relative metabolite abundance was expressed as normalized peak area. Differential metabolites were screened using a two-step strategy. First, metabolites differing between the 2013 and 2023 samples were selected based on VIP ≥ 1.0 from OPLS-DA, |log_2_fold change| ≥ 1, and false discovery rate (FDR)-adjusted *p* < 0.05. FDR correction was performed using the Benjamini–Hochberg procedure. Second, Spearman correlation analysis was then performed on the primary differential metabolites to identify candidate storage-associated metabolites showing monotonic relationships with storage duration across the five representative production years. Metabolites with |Spearman r| ≥ 0.9 and *p* < 0.05 were retained as storage-associated metabolites. This screening strategy was used for candidate discovery within the present dataset.

### 2.6 Antioxidant activity analysis

Free radical scavenging activities against DPPH·, ·OH, ${{\text{O}}_{2}}^{-}\cdot$, and PTIO· were determined using freshly prepared tea infusions. Briefly, 1.500 g of ground tea sample was extracted with 200 mL of boiling distilled water for 30 min in a boiling water bath, with intermittent shaking every 10 min. The infusion was vacuum-filtered while hot, cooled to room temperature, and diluted to 250 mL with distilled water. The prepared infusion was further diluted as required for each assay. All measurements were performed using three biological replicates. For statistical analysis and visualization, scavenging rates were expressed as decimal values.

#### 2.6.1 DPPH· scavenging activity

DPPH· scavenging activity was determined according to the method of Brand-Williams et al. (1995) with minor modifications. The tea infusion was diluted 80-fold with distilled water before analysis. Briefly, 2 mL of diluted tea infusion was mixed with 2 mL of freshly prepared DPPH· ethanolic solution (0.06 mg/mL), incubated in the dark at room temperature for 20 min, and measured at 517 nm using a UV–visible spectrophotometer (UV-1800PC, Meipuda, Shanghai, China). DPPH· scavenging activity was calculated as follows:


$$\text{Scavenging rate}\left(\%\right)=[1-({\text{A}}_{\text{i}}-{\text{A}}_{\text{j}})/{\text{A}}_{0}]\times 100\%$$

where A_0_ is the absorbance of the control, A_i_ is the absorbance of the sample reaction mixture, and A_j_ is the absorbance of the sample blank.

#### 2.6.2 ·OH scavenging activity

Hydroxyl radical (·OH) scavenging activity was determined using a commercial assay kit (Cat. No. A018-1-1; Nanjing Jiancheng Bioengineering Institute, Nanjing, China) based on the Fenton reaction and Griess color development, following the manufacturer’s instructions with minor modifications (Smirnoff and Cumbes, 1989). The tea infusion was diluted 30-fold before analysis. Absorbance was measured at 550 nm, and ·OH scavenging activity was calculated as follows:


$$\text{Scavenging rate}\left(\%\right)=\left[1-\left({\text{A}}_{\text{sample}}-{\text{A}}_{\text{blank}}\right)/{\text{A}}_{\text{control}}\right]\times 100\%$$

where A_control_ is the absorbance of the control reaction, A_sample_ is the absorbance of the sample group, and A_blank_ is the absorbance of the reagent blank.

#### 2.6.3 ${{\text{O}}_{2}}^{-}\cdot$ scavenging activity

Superoxide anion radical ( ${{\text{O}}_{2}}^{-}\cdot$) scavenging activity was determined using a commercial assay kit based on the xanthine oxidase–hydroxylamine method (Cat. No. A001-1; Nanjing Jiancheng Bioengineering Institute, Nanjing, China), following the manufacturer’s instructions (Elstner and Heupel, 1976). The tea infusion was diluted 5-fold before analysis. Absorbance was measured at 550 nm, and ${{\text{O}}_{2}}^{-}\cdot$ scavenging activity was calculated as follows:


$$\text{Scavenging rate}\left(\%\right)=\left[\left({\text{A}}_{\text{control}}-{\text{A}}_{\text{sample}}\right)/{\text{A}}_{\text{control}}\right]\times 100\%$$

where A_control_ and A_sample_ are the absorbances of the control and sample groups, respectively.

#### 2.6.4 PTIO· scavenging activity

PTIO· scavenging activity was determined according to a previously described method with minor modifications (Li, 2017). PTIO· solution was prepared at a concentration of 0.1 mg/mL. For the assay, 0.5 mL of tea infusion was mixed with 3.5 mL of PTIO· solution, incubated at 37 °C for 2 h in the dark, and measured at 557 nm. Distilled water was used to prepare the control and sample blank. PTIO scavenging activity was calculated as follows:


$$\text{Scavenging rate}\left(\%\right)=\left[1-\left({\text{A}}_{1}-{\text{A}}_{2}\right)/{\text{A}}_{0}\right]\times 100\%$$

where A_0_ is the absorbance of the control, A_1_ is the absorbance of the sample reaction mixture, and A_2_ is the absorbance of the sample blank.

### 2.7 Statistical analysis and data integration

All statistical analyses were performed using Python 3.9 with SciPy v1.11.1 (Virtanen et al., 2020), scikit-learn v1.3.0 (Pedregosa et al., 2011), pandas v2.0.3 (McKinney, 2010), NumPy v1.24.3 (Harris et al., 2020), and Matplotlib v3.7.1 (Hunter, 2007). Statistical significance was defined as *p* < 0.05 unless otherwise stated. Significance levels were denoted as *p < 0.05, **p < 0.01, and ***p < 0.001.

#### 2.7.1 Univariate statistical analysis

Data are presented as mean ± standard deviation (SD) of three biological replicates unless otherwise stated. One-way analysis of variance (ANOVA) was used to evaluate differences among production years, followed by Tukey’s honestly significant difference (HSD) test for *post-hoc* comparisons. Different lowercase letters were used to indicate significant differences among groups at *p* < 0.05. Spearman rank correlation analysis was used to assess monotonic relationships between individual variables and storage duration. Pearson correlation analysis was used to evaluate linear associations between continuous variables.

#### 2.7.2 Multivariate statistical analysis

Principal component analysis (PCA) was performed to visualize sample clustering and multivariate variation. Before PCA, variables were mean-centered and scaled to unit variance using Z-score normalization. The first two principal components were used for visualization, and 95% confidence ellipses were calculated for sample groups where applicable. Orthogonal partial least squares discriminant analysis (OPLS-DA) was used to evaluate metabolomic separation among storage years and to identify metabolites contributing to group discrimination (Trygg and Wold, 2002). OPLS-DA was performed using MetaboAnalyst 6.0 (Pang et al., 2022). Model performance was assessed using *R^2^X*, *R^2^Y*, and *Q^2^* values, and model robustness was evaluated using 200 permutation tests. Metabolites with VIP ≥ 1.0, fold change ≥ 2.0 or ≤ 0.5, and *p* < 0.05 were considered primary differential metabolites.

#### 2.7.3 Multi-modal data integration

Multi-modal integration was performed using 19 key variables selected from five analytical modalities: sensory attributes, electronic tongue responses, chemical components, storage-associated metabolites, and antioxidant activities. The variables included Overall score, Liquor color, Sour, Smooth, Brisk, electronic tongue Sourness, Bitterness, and Astringency, Polyphenols, Theaflavins, Thearubigins, Theabrownins, LysoPC(19:2), Abietic acid, Dehydro-EGC-Glc-Ara, Hydroxyricinoleic acid, PTIO· scavenging, ·OH scavenging, and DPPH· scavenging. These analyses were performed using year means from the five production years with complete multi-modal datasets: 2013, 2016, 2019, 2022, and 2023. All variables were Z-score standardized before integration.

A Pearson correlation matrix was constructed to evaluate pairwise associations among the 19 variables. Partial least squares regression (PLSR) was then used to predict storage duration from the 19 variables, using storage duration in years as the response variable. The optimal number of latent variables was selected by leave-one-out cross-validation (LOOCV), testing one to four latent variables. Model performance was evaluated using training *R^2^*, training RMSE, LOOCV *Q^2^*, and RMSECV. Variable importance in projection (VIP) scores were calculated according to Chong and Jun (2005), and variables with VIP > 1.0 were considered important contributors to the PLSR model.

To provide an interpretable index for storage-related quality evaluation, a composite storage score was calculated across all eight production years. Seven core storage-associated indicators were selected, including Sour, Theabrownins, Smooth, Brisk, PTIO· scavenging, Liquor color, and Theaflavins. Each variable was Z-score standardized across the eight-year dataset before score calculation. The composite score was calculated as follows:


Composite Score=+0.25×Z(Sour)+0.20×Z(Theabrownins)+0.10×Z(Smooth)−0.15×Z(Brisk)−0.15×Z(PTIO· scvenging)−0.10×Z(Liquor color)−0.05×Z(Theaflavins)

where Z(x) denotes the Z-score-standardized value of variable x. Positive weights were assigned to aged-tea-associated indicators, whereas negative weights were assigned to fresh-tea-associated or storage-depleted indicators. The weighting scheme was defined based on expert selection and biological interpretability, supported by the univariate storage-association patterns and PLSR variable importance results. The weights were not empirically optimized and were used to construct a descriptive index for summarizing storage-associated quality variation within the present dataset. Raw composite scores were linearly normalized to a 0–10 scale, with the lowest raw score set to 0 and the highest raw score set to 10. Based on the normalized score, samples were classified into three storage stages: Fresh (0–3.3), Intermediate (3.3–6.7), and Aged (6.7–10). Concordance between the composite score and actual storage duration was evaluated using Spearman and Pearson correlation analyses.

## 3 Results

### 3.1 Visual and sensory characterization reveals attribute-specific responses to storage

**Figure 1:**
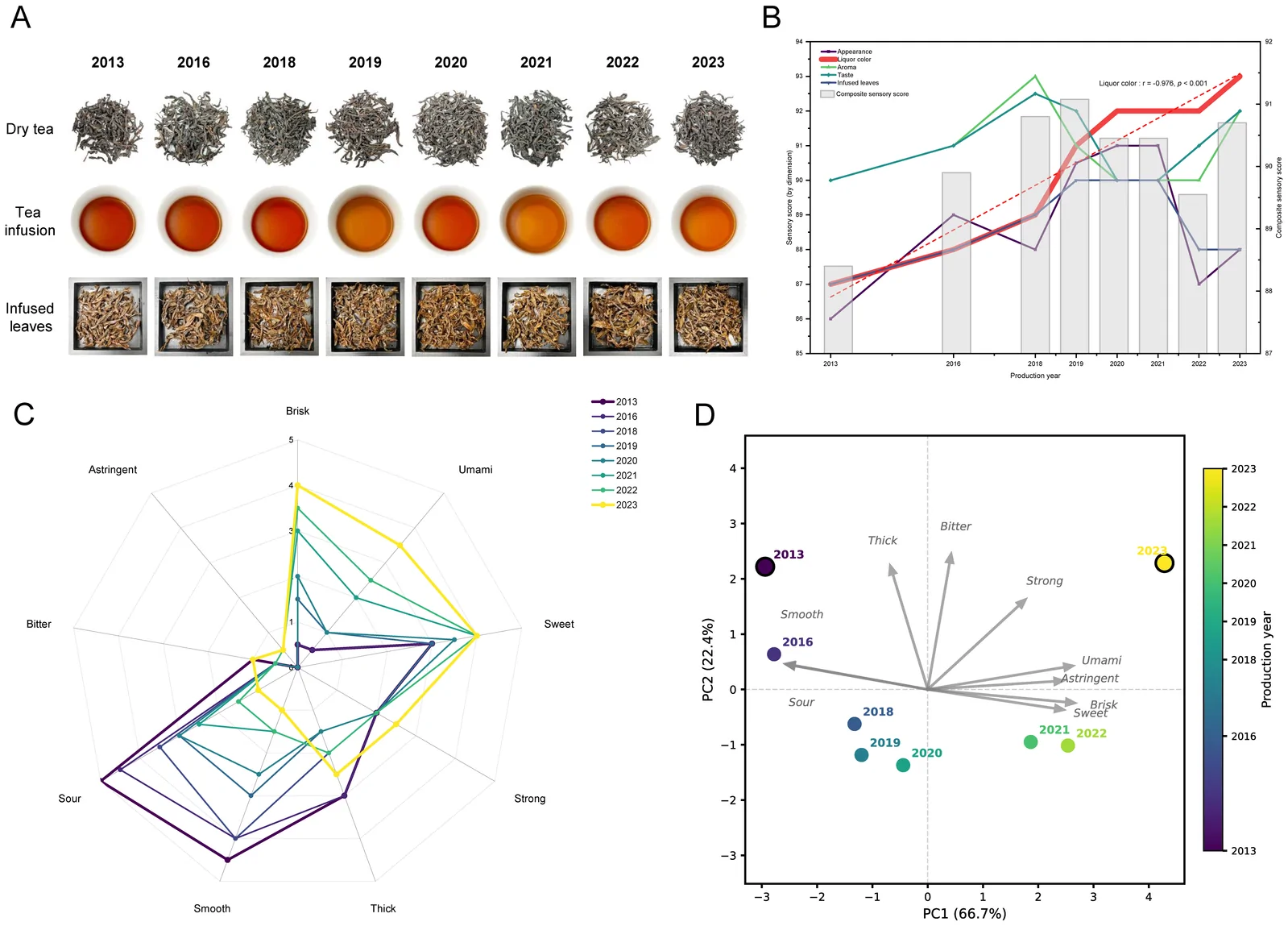
Visual documentation and sensory profiling of Lapsang Souchong black tea samples during storage. **(A)** Representative images of dry tea leaves, tea infusions, and infused leaves from eight production years (2013, 2016, 2018, 2019, 2020, 2021, 2022, and 2023). **(B)** Traditional sensory evaluation scores for five quality attributes and the corresponding total scores. The dashed line indicates the fitted trend for liquor color, which was significantly correlated with storage duration (Spearman r = −0.976, *p* < 0.001). **(C)** Radar plot of quantitative descriptive analysis (QDA) based on nine taste attributes. Samples are color-coded according to production year, and the longest-stored and freshest samples are highlighted. Storage-responsive attributes included Sour, Smooth, Brisk, Umami, Sweet, and Astringent. **(D)** PCA biplot based on Z-score-standardized QDA data. Points represent samples, and arrows indicate QDA attribute loadings. The distribution of samples along PC1 reflects the storage-related sensory evolution from fresh to aged tea.

The eight Lapsang Souchong samples produced between 2013 and 2023 were first characterized by visual documentation, traditional sensory evaluation, and quantitative descriptive analysis (QDA) (**Figure 1**). Photographic documentation of dry tea leaves, tea infusions, and infused leaves revealed clear storage-related visual differences, especially in tea infusion color (**Figure 1A**). The infusion changed from a deep orange-red hue in the longest-stored samples (2013 and 2016) to a bright orange-yellow appearance in the freshest samples (2022 and 2023), whereas dry leaves and infused leaves showed comparatively subtle differences. This suggested that infusion color was the most visually sensitive attribute affected by long-term storage in Lapsang Souchong. Consistently, traditional sensory evaluation showed that liquor color was the only sensory dimension with a significant monotonic relationship with storage duration (Spearman r = −0.976, *p* < 0.001), decreasing from 93 in the freshest sample to 87 in the ten-year-stored sample (**Figure 1B**). In contrast, the total sensory scores remained within a narrow range of 88.4–91.1 and did not show a monotonic decline, indicating that all samples maintained relatively high overall sensory quality. The other sensory dimensions, including appearance, aroma, taste, and infused leaves, showed no significant monotonic trends with storage duration.

To further resolve taste changes that may be masked by total sensory scores, QDA was performed for nine taste attributes (**Figure 1C**). Six attributes showed significant monotonic relationships with storage duration. Sour (Spearman r = +0.994, *p* < 0.0001) and Smooth (r = +0.988, *p* < 0.0001) increased progressively with storage, whereas Brisk (r = −0.988, *p* < 0.0001), Umami (r = −0.970, *p* < 0.0001), Sweet (r = −0.882, *p* = 0.004), and Astringent (r = −0.845, *p* = 0.008) decreased. The remaining attributes, including Strong, Thick, and Bitter, remained relatively stable across production years. These results indicated that storage induced a bidirectional taste transformation, characterized by the accumulation of aged-tea-associated attributes and the attenuation of fresh-tea-associated attributes. PCA based on Z-score-standardized QDA data further supported this sensory trajectory (**Figure 1D**). The first two principal components explained 89.1% of the total variance, with PC1 and PC2 accounting for 66.7% and 22.4%, respectively. The eight samples were distributed continuously along PC1, with the longest-stored sample and the freshest sample located at opposite ends. Loading vectors separated Sour and Smooth from Brisk, Umami, Sweet, and Astringent along PC1, indicating that PC1 mainly represented the storage-related transition from fresh-tea taste characteristics to aged-tea taste characteristics.

Overall, the visual, traditional sensory, QDA, and PCA results consistently showed that long-term storage did not cause a uniform decline in sensory quality. Instead, storage reshaped the sensory profile of Lapsang Souchong in an attribute-specific manner, with increased Sour and Smooth and decreased Brisk, Umami, and Sweet. These findings provide a sensory basis for further investigating the chemical and metabolic factors underlying storage-related quality transformation.

### 3.2 Chemical basis of storage-associated changes: theabrownin accumulation and reduced TR/TB ratio as key indicators

**Figure 2:**
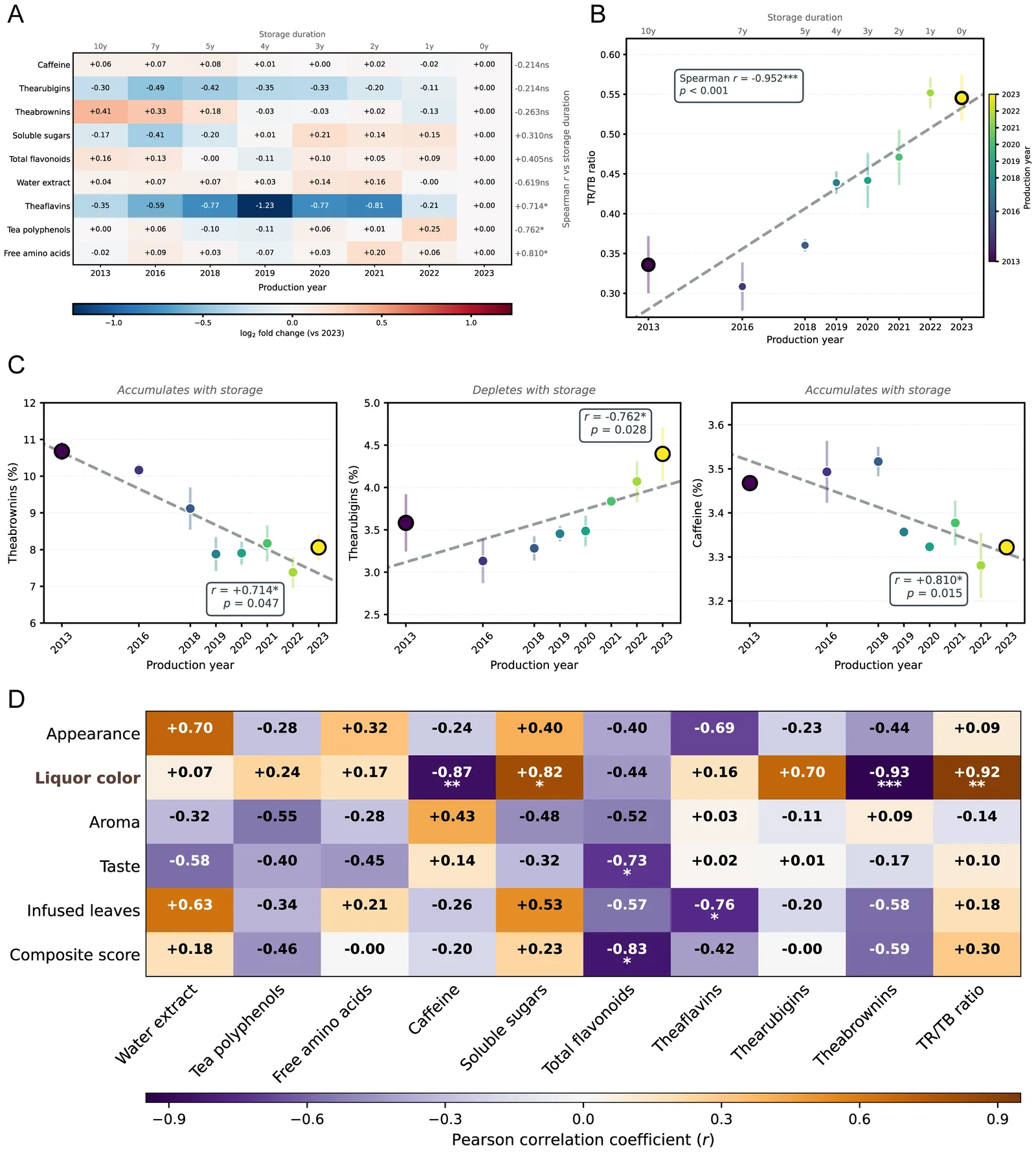
Storage-related changes in taste-related chemical components of Lapsang Souchong black tea. **(A)** Heatmap showing the log_2_fold changes of nine chemical components in each production year relative to the 2023 sample. Rows are ordered by the absolute Spearman correlation coefficient between component content and storage duration. **(B)** Changes in the thearubigin-to-theabrownin ratio (TR/TB) across eight production years. The dashed line indicates the fitted trend, and the Spearman correlation coefficient is shown in the panel. **(C)** Temporal changes in theabrownins, thearubigins, and caffeine, which showed significant correlations with storage duration. Dashed lines indicate fitted trends. **(D)** Pearson correlation heatmap between sensory evaluation attributes and chemical components. Color intensity represents Pearson correlation coefficients, and significance levels are indicated in each cell. Data are shown as mean ± SD (n = 3). Significance levels: *p < 0.05, **p < 0.01, ***p < 0.001; ns, not significant.

To investigate the chemical basis of the sensory changes described above, nine taste-related chemical components were quantified across the eight Lapsang Souchong samples. Their contents, together with one-way ANOVA and Tukey’s HSD *post-hoc* test results, are summarized in **Table 1**, and their dynamic patterns and sensory associations are shown in **Figure 2**. One-way ANOVA showed significant differences among production years for all nine chemical components, with F values ranging from 3.36 for water extract (*p* = 0.021) to 98.11 for soluble sugars (*p* < 0.001) (**Table 1**). These results indicated that long-term storage broadly affected the taste-related chemical composition of Lapsang Souchong. However, Spearman correlation analysis revealed that only three components showed significant monotonic relationships with storage duration (**Figure 2A**). Theabrownins (TB; Spearman r = +0.714, *p* = 0.047) and caffeine (r = +0.810, *p* = 0.015) increased with storage duration, whereas thearubigins (TR; r = −0.762, *p* = 0.028) decreased. The other components, including soluble sugars, free amino acids, polyphenols, theaflavins, water extract, and total flavonoids, varied significantly among years but did not show consistent monotonic trends. This suggests that storage-associated chemical changes in Lapsang Souchong include both time-dependent trends and year-specific fluctuations. The temporal trajectories of TB, TR, and caffeine are shown in **Figure 2C**. TB content was higher in the long-stored samples, reaching 10.68 ± 0.25% in 2013 and 10.17 ± 0.16% in 2016, but decreased to 7.38–8.17% in samples stored for less than five years. In contrast, TR content was lower in long-stored samples and increased toward the fresher samples, from 3.13 ± 0.26% in 2016 to 4.40 ± 0.32% in 2023. Caffeine showed a modest but significant positive association with storage duration, ranging from 3.28–3.32% in the freshest samples to 3.47–3.52% in the long-stored samples. As a result of the opposite trends of TB and TR, the TR/TB ratio was lower in aged samples and higher in fresh samples, increasing from 0.31 in 2016 to approximately 0.55 in 2022–2023 (**Figure 2B**). Accordingly, the TR/TB ratio was negatively correlated with storage duration, indicating that a lower TR/TB ratio reflected a more aged chemical profile. This pattern is consistent with the progressive conversion and polymerization of tea pigments during black tea storage, in which lower-molecular-weight pigments are gradually transformed into more polymerized brown pigments.

**Table 1:** Sample information and contents of taste-related chemical components of Lapsang Souchong black tea samples produced between 2013 and 2023.

Sample	Storage (years)	Water extract (%)	Tea polyphenols (%)	Free amino acids (%)	Caffeine (%)	Soluble sugars (%)	Total flavonoids (mg/g)	Theaflavins (%)	Thearubigins (%)	Theabrownins (%)
2013	10	30.89 ± 1.61 ^ab^	7.56 ± 0.32 ^bc^	2.35 ± 0.03 ^cd^	3.47 ± 0.02 ^a^	2.97 ± 0.04 ^c^	14.43 ± 0.31 ^a^	0.227 ± 0.004 ^b^	3.58 ± 0.34 ^b^	10.68 ± 0.25 ^a^
2016	7	31.46 ± 0.85 ^ab^	7.86 ± 0.07 ^b^	2.54 ± 0.05 ^b^	3.49 ± 0.07 ^a^	2.52 ± 0.01 ^d^	14.10 ± 0.35 ^a^	0.192 ± 0.004 ^c^	3.13 ± 0.26 ^b^	10.17 ± 0.16 ^a^
2018	5	31.39 ± 0.44 ^ab^	7.05 ± 0.14 ^c^	2.43 ± 0.03 ^bc^	3.52 ± 0.03 ^a^	2.92 ± 0.03 ^c^	12.89 ± 0.10 ^b^	0.170 ± 0.018 ^c^	3.28 ± 0.14 ^b^	9.12 ± 0.58 ^b^
2019	4	30.65 ± 1.30 ^ab^	6.98 ± 0.10 ^c^	2.27 ± 0.02 ^d^	3.36 ± 0.01 ^b^	3.39 ± 0.15 ^b^	11.96 ± 0.33 ^c^	0.124 ± 0.018 ^d^	3.45 ± 0.09 ^b^	7.88 ± 0.46 ^c^
2020	3	33.14 ± 0.66 ^ab^	7.87 ± 0.09 ^b^	2.44 ± 0.03 ^bc^	3.32 ± 0.00 ^b^	3.87 ± 0.01 ^a^	13.82 ± 0.24 ^a^	0.170 ± 0.017 ^c^	3.49 ± 0.18 ^b^	7.90 ± 0.31 ^c^
2021	2	33.52 ± 0.22 ^a^	7.59 ± 0.41 ^b^	2.75 ± 0.08 ^a^	3.38 ± 0.05 ^b^	3.70 ± 0.08 ^a^	13.37 ± 0.33 ^b^	0.165 ± 0.015 ^c^	3.84 ± 0.05 ^a^	8.17 ± 0.49 ^bc^
2022	1	29.96 ± 1.37 ^b^	8.96 ± 0.10 ^a^	2.49 ± 0.03 ^b^	3.28 ± 0.07 ^b^	3.74 ± 0.13 ^a^	13.76 ± 0.17 ^a^	0.251 ± 0.007 ^b^	4.07 ± 0.24 ^a^	7.38 ± 0.41 ^c^
2023	0	29.99 ± 2.26 ^ab^	7.55 ± 0.19 ^bc^	2.39 ± 0.02 ^c^	3.32 ± 0.01 ^b^	3.36 ± 0.08 ^b^	12.91 ± 0.40 ^b^	0.289 ± 0.004 ^a^	4.40 ± 0.32 ^a^	8.06 ± 0.35 ^bc^

Pearson correlation analysis was further performed to examine the relationships between chemical components and traditional sensory evaluation attributes (**Figure 2D**). Among the sensory dimensions, liquor color showed the strongest associations with chemical composition. It was significantly correlated with TB (r = −0.93, *p* < 0.001), the TR/TB ratio (r = +0.92, *p* < 0.001), caffeine (r = −0.87, *p* < 0.001), and soluble sugars (r = +0.82, *p* < 0.05). In contrast, appearance, aroma, taste, infused leaves, and the total sensory score showed no significant correlations with the measured chemical components. These results indicate that pigment-related changes, especially TB accumulation and the decline in the TR/TB ratio, are closely associated with the storage-related change in liquor color observed in **Figure 1**.

Several non-pigment components also displayed marked year-to-year variation. Soluble sugars showed the largest variation among samples (F = 98.11, *p* < 0.001), ranging from 2.52 ± 0.01% in 2016 to 3.87 ± 0.01% in 2020, but without a monotonic storage-related trend. Total polyphenols were highest in 2022 and lowest in 2018–2019, suggesting moderate fluctuations rather than a consistent storage response. Theaflavins also showed a non-monotonic pattern, with the lowest level in 2019 and the highest level in 2023. Together, these results suggest that the chemical evolution of stored Lapsang Souchong is not governed solely by linear pigment oxidation but also involves more complex compositional changes. This provided the rationale for further metabolomic profiling to identify broader molecular changes associated with long-term storage.

### 3.3 Electronic tongue analysis provides instrumental validation of storage-associated taste shifts

**Figure 3:**
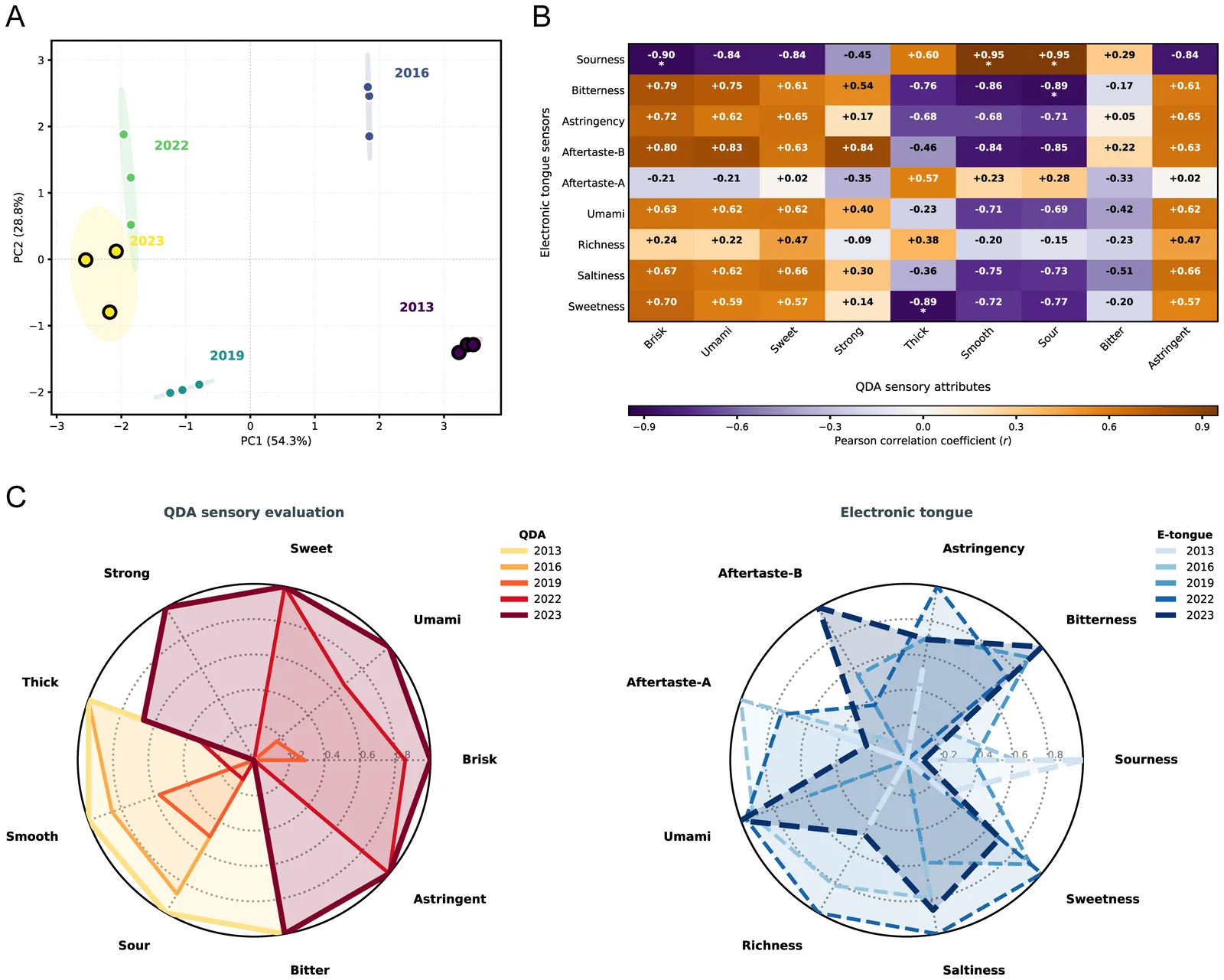
Electronic tongue profiling of stored Lapsang Souchong black tea and its relationship with QDA sensory attributes. **(A)** PCA score plot based on nine electronic tongue sensor responses from five representative production years. Each point represents one replicate measurement (n = 3), and ellipses indicate 95% confidence regions. **(B)** Pearson correlation heatmap between electronic tongue responses and QDA attributes. Gold-highlighted cells indicate conceptually matched sensory–instrumental attribute pairs. Electronic tongue Sourness showed a significant positive correlation with sensory Sour (r = +0.95, *p* = 0.011). Significance levels: *p < 0.05, **p < 0.01, ***p < 0.001. **(C)** Radar plots comparing min-max-normalized QDA sensory attributes and electronic tongue responses.

To complement the QDA assessment with an objective instrument-based measurement, electronic tongue analysis was performed on five representative samples spanning the storage gradient: 2013, 2016, 2019, 2022, and 2023. Nine taste-related sensor responses were recorded, including Sourness, Bitterness, Astringency, Aftertaste-B, Aftertaste-A, Umami, Richness, Saltiness, and Sweetness. The resulting data were analyzed by PCA for sample discrimination and by Pearson correlation analysis against QDA attributes for cross-modal validation (**Figure 3**).

PCA based on the nine electronic tongue responses separated the five production years into distinct clusters in the PC1–PC2 space (**Figure 3A**). The samples were distributed continuously along PC1, with the longest-stored sample (2013) and the freshest sample (2023) located at opposite ends, while the intermediate samples were positioned between them. The largely separated 95% confidence ellipses indicated that electronic tongue profiling could distinguish samples from different storage years. This pattern was broadly consistent with the QDA-based PCA results (**Figure 1D**), suggesting that the storage-related taste changes identified by sensory evaluation were also captured by instrumental taste profiling.

To compare the two modalities at the overall profile level, min–max-normalized QDA scores and electronic tongue responses were visualized using radar plots (**Figure 3C**). Both approaches showed clear storage-related differences. The long-stored samples were more closely associated with aged-tea sensory features such as Sour and Smooth in the QDA profile, while fresh samples were more associated with fresh-tea attributes such as Brisk, Umami, and Sweet. The 2019 sample showed an intermediate profile, consistent with its position in the storage gradient. These results indicate that QDA and electronic tongue analysis provided broadly consistent descriptions of storage-associated taste evolution, despite their different measurement principles and response scales.

Pearson correlation analysis was further used to evaluate attribute-level concordance between electronic tongue responses and QDA attributes (**Figure 3B**). Among the conceptually matched pairs, including Sourness–Sour, Bitterness–Bitter, Astringency–Astringent, Umami–Umami, and Sweetness–Sweet, the Sourness–Sour pair showed the strongest and only significant correlation (r = +0.95, *p* = 0.011). This result supports the reliability of sourness as a storage-responsive taste attribute and is consistent with its strong monotonic association with storage duration in the QDA analysis (**Figure 1C**). Other matched pairs showed moderate but non-significant correlations or weak correlations, which may be partly attributed to the limited number of samples and to the complex physicochemical basis of bitterness, astringency, umami, and sweetness perception. In addition to the matched attribute pairs, several cross-attribute correlations further reflected the storage-related taste gradient. Electronic tongue Sourness was positively correlated with QDA Smooth (r = +0.95, *p* = 0.014) and negatively correlated with QDA Brisk (r = −0.90, *p* = 0.036), indicating that instrumental sourness was associated not only with perceived sourness but also with the broader transition from fresh-tea to aged-tea taste characteristics. Electronic tongue Bitterness was negatively correlated with QDA Sour (r = −0.89, *p* = 0.043), and electronic tongue Sweetness was negatively correlated with QDA Thick (r = −0.89, *p* = 0.044). These correlations suggest that electronic tongue responses captured aspects of the overall chemical structure of taste evolution, even when individual sensor labels did not correspond directly to QDA attribute names.

Overall, electronic tongue analysis provided instrumental support for the storage-associated taste shifts observed by QDA. The PCA results distinguished samples along the storage gradient, and the correlation analysis highlighted Sourness as the most consistent cross-modal attribute. These findings indicate that the taste evolution of stored Lapsang Souchong is a measurable and reproducible phenomenon, providing further support for subsequent metabolomic analysis of the molecular basis of storage-related quality transformation.

### 3.4 Metabolomic profiling reveals storage-associated compositional reorganization

**Figure 4:**
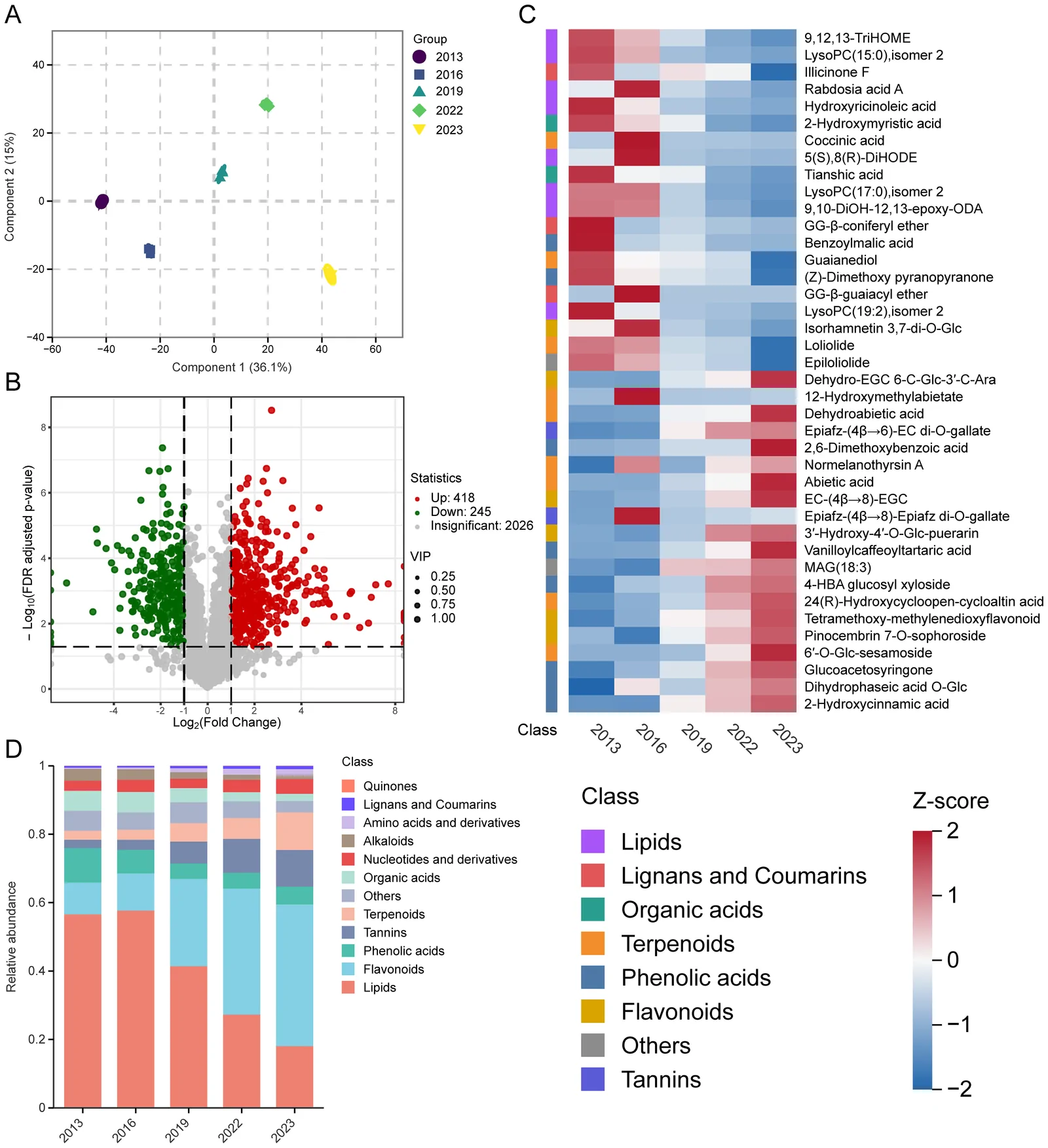
Metabolomic profiling reveals storage-associated molecular remodeling in Lapsang Souchong black tea. **(A)** OPLS-DA score plot based on widely targeted metabolomic data from five representative production years. Each point represents one biological replicate (n = 3), and ellipses indicate 95% confidence regions. Model parameters were *R^2^X* = 0.511, *R^2^Y* = 0.996, and *Q^2^* = 0.979. **(B)** Volcano plot showing differential metabolites between the longest-stored sample (2013) and the freshest sample (2023). Significant metabolites were selected using VIP ≥ 1, |log_2_fold change| ≥ 1, and FDR-adjusted *p* < 0.05. **(C)** Z-score-normalized heatmap of the top 40 storage-associated metabolites. The upper and lower groups represent metabolites positively and negatively correlated with storage duration, respectively. **(D)** Stacked bar plot showing the compound class distribution of 183 storage-associated metabolites selected using differential abundance analysis and Spearman correlation analysis.

To obtain a molecular-level view of the storage-related changes suggested by the sensory, electronic tongue, and chemical analyses, widely targeted UPLC–MS/MS metabolomics was performed on five representative samples: 2013, 2016, 2019, 2022, and 2023, with three biological replicates per sample. A total of 2,694 metabolites were detected and quantified across all samples, covering 12 chemical classes, including lipids, flavonoids, tannins, phenolic acids, terpenoids, alkaloids, organic acids, nucleotides and derivatives, amino acids and derivatives, and lignans and coumarins. OPLS-DA showed clear separation among the five representative production years, with good model performance parameters (*R^2^X* = 0.512, *R^2^Y* = 0.996, *Q^2^* = 0.979; **Figure 4A**). Model robustness was further supported by 200 permutation tests, in which the original *R^2^Y* and *Q^2^* values were higher than those obtained from the permuted models, indicating that the OPLS-DA model was not generated by random class separation (**Supplementary Figure 1**). The samples were distributed along Component 1 according to storage duration, with the longest-stored samples located on one side of the score plot and the freshest samples on the opposite side. The tight clustering of biological replicates within each production year indicated good analytical reproducibility. This metabolomic separation was consistent with the sensory, electronic tongue, and chemical trends described above, suggesting that storage-associated quality changes were accompanied by systematic molecular reorganization.

To identify metabolites associated with storage, a two-step filtering strategy was applied. First, the comparison between the longest-stored sample (2013) and the freshest sample (2023) identified 663 differential metabolites using the criteria VIP ≥ 1, |log_2_fold change| ≥ 1, and FDR-adjusted *p* < 0.05 (**Figure 4B**; **Supplementary Table 9**). Among them, 245 metabolites were more abundant in the 2013 sample, whereas 418 metabolites were more abundant in the 2023 sample. Second, these differential metabolites were further filtered by Spearman correlation with storage duration across the five samples. Using |r| ≥ 0.9 and *p* < 0.05,183 storage-associated metabolites were retained, including 100 positively correlated and 83 negatively correlated metabolites (**Supplementary Table 1**). These metabolites therefore represented compounds that not only differed between the storage extremes but also changed consistently across the storage gradient. The top 40 storage-associated metabolites were visualized by heatmap analysis, including 20 storage-accumulating and 20 storage-depleting markers (**Figure 4C**). Storage-accumulating metabolites were mainly composed of lipid-derived and oxidized fatty-acid-related compounds, including LysoPC(19:2), 9,12,13-TriHOME, LysoPC(15:0), LysoPC(17:0), hydroxyricinoleic acid, 2-hydroxymyristic acid, 5(S),8(R)-DiHODE, tianshic acid, and 9,10-DiOH-12,13-epoxy-ODA. The accumulation of these compounds suggests that lipid hydrolysis and oxidative transformation were important molecular processes during long-term storage of Lapsang Souchong black tea. In addition, several terpenoid- or norisoprenoid-related metabolites, such as illicinone F, rabdosia acid A, coccinic acid, guaianediol, loliolide, and epiloliolide, together with lignan derivatives such as GG-β-coniferyl ether and GG-β-guaiacyl ether, also accumulated in aged samples, indicating that multiple oxidative and structural rearrangement processes occurred during storage. In contrast, storage-depleting metabolites were enriched in flavonoid-, tannin-, phenolic acid-, and diterpenoid-related compounds. Representative compounds included dehydro-EGC 6-C-Glc-3′-C-Ara, epiafzelechin-(4β→6)-EC di-O-gallate, EC-(4β→8)-EGC, epiafzelechin-(4β→8)-epiafzelechin di-O-gallate, 3′-hydroxy-4′-O-Glc-puerarin, pinocembrin 7-O-sophoroside, tetramethoxy-methylenedioxyflavonoid, vanilloylcaffeoyltartaric acid, 4-HBA glucosyl xyloside, and 2-hydroxycinnamic acid. The depletion of these flavonoid-, tannin-, and phenolic acid-related metabolites was consistent with the progressive transformation of polyphenolic precursors during storage. Notably, several pine-resin-related diterpenoids, including abietic acid, dehydroabietic acid, and 12-hydroxymethylabietate, were markedly depleted in aged samples. Their decline suggests that diterpenoid compounds associated with pinewood smoking or resinous characteristics may gradually diminish during long-term storage, potentially contributing to the mellowing of the smoky and resinous profile of Lapsang Souchong black tea.

The compound-class distribution of the 183 storage-associated metabolites further revealed broad compositional shifts across the storage gradient (**Figure 4D**). Lipids accounted for the largest proportion in the longest-stored sample, representing approximately 56.6% of the metabolite profile in 2013, but decreased to 18.0% in the freshest sample from 2023. In contrast, flavonoids increased markedly from 9.3% in the 10-year-stored sample to 41.4% in the fresh sample, becoming the dominant class in 2023. Tannins and terpenoids also showed higher proportions in fresher samples, increasing from 2.4% to 10.7% and from 2.7% to 11.0%, respectively, from 2013 to 2023. By contrast, phenolic acids, organic acids, and alkaloids were relatively enriched in long-stored samples, decreasing from 10.1%, 5.8%, and 3.5% in 2013 to 5.2%, 2.1%, and 1.1% in 2023, respectively. Minor classes, including nucleotides and derivatives, amino acids and derivatives, lignans and coumarins, and quinones, accounted for relatively small proportions but also contributed to the overall compositional variation. Overall, these results indicate that long-term storage of Lapsang Souchong black tea is accompanied by systematic class-level metabolic reorganization, characterized mainly by the relative enrichment of lipid-derived metabolites and phenolic acids in long-stored samples, together with the depletion of flavonoids, tannins, and terpenoids. These class-level shifts provide molecular evidence for the chemical transformation and sensory changes observed during storage.

### 3.5 Antioxidant activity analysis reveals radical-specific storage responses and highlights PTIO· scavenging as a storage-responsive functional indicator

**Figure 5:**
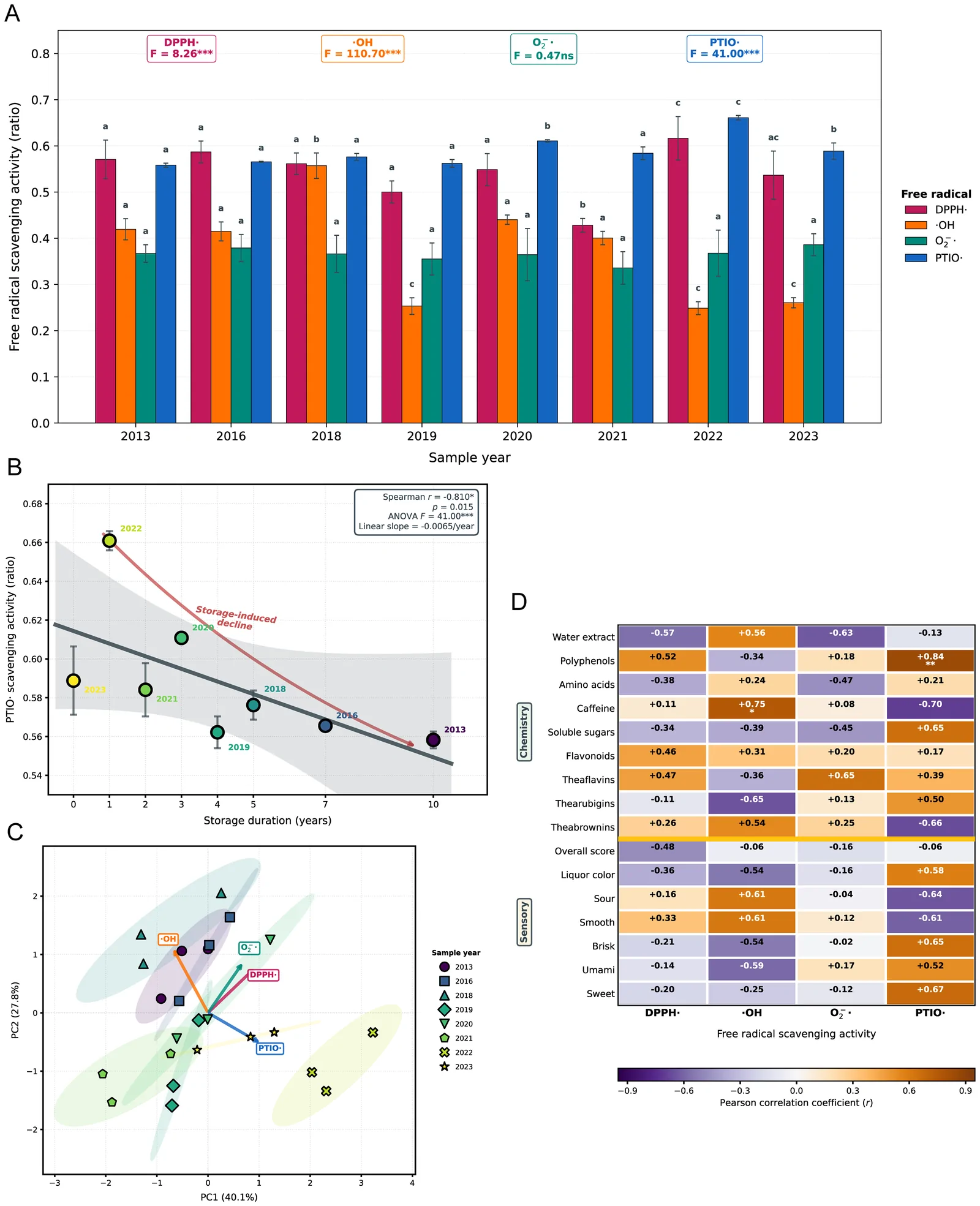
Antioxidant activity profiling of Lapsang Souchong black tea during storage. **(A)** Free radical scavenging activities against DPPH·, ·OH, ${{\text{O}}_{2}}^{-}\cdot$, and PTIO· across eight production years. Bars represent mean ± SD (n = 3). Different lowercase letters indicate significant differences among years within each assay according to Tukey’s HSD test (*p* < 0.05). **(B)** Relationship between PTIO· scavenging activity and storage duration. Points represent mean ± SD (n = 3), and the shaded area indicates the 95% confidence interval of the fitted regression line. Spearman correlation coefficient and *p* value are shown in the panel. **(C)** PCA biplot based on the four free radical scavenging activities. Points represent biological replicates, ellipses indicate 95% confidence regions, and arrows represent antioxidant assay loadings. **(D)** Pearson correlation heatmap between free radical scavenging activities and selected chemical and sensory attributes. Correlations were calculated using year means (n = 8). Color intensity represents Pearson correlation coefficients, and significance levels are indicated by asterisks. Significance levels: *p < 0.05, **p < 0.01, ***p < 0.001; ns, not significant.

To evaluate the functional consequences of long-term storage, the antioxidant activities of the eight Lapsang Souchong samples were assessed using four free radical scavenging assays, including DPPH·, ·OH, ${{\text{O}}_{2}}^{-}\cdot$, and PTIO·. These assays were selected to represent different antioxidant response mechanisms and to determine whether storage affected antioxidant activity in a radical-specific manner. The four assays showed distinct responses to storage (**Figure 5A**). One-way ANOVA revealed significant differences among production years for DPPH· scavenging (F = 8.26, *p* < 0.001), ·OH scavenging (F = 110.70, *p* < 0.001), and PTIO· scavenging (F = 41.00, *p* < 0.001), whereas ${{\text{O}}_{2}}^{-}\cdot$ scavenging remained relatively stable (F = 0.47, *p* = 0.841). Among the assays, ·OH scavenging showed the largest variation, ranging from 0.249 in 2022 to 0.557 in 2018. In contrast, ${{\text{O}}_{2}}^{-}\cdot$ scavenging varied only slightly across samples. These results indicate that the antioxidant response of Lapsang Souchong to storage is radical-specific rather than uniform. Among the four assays, PTIO· scavenging showed a significant negative association with storage duration (Spearman r = −0.810, *p* = 0.015; **Figure 5B**). PTIO· scavenging was generally higher in the fresher samples and lower in the long-stored samples, with the lowest value observed in the 2013 sample. This trend differed from the more irregular patterns observed for DPPH·, ·OH, and ${{\text{O}}_{2}}^{-}\cdot$ scavenging. Thus, PTIO· scavenging may serve as a storage-responsive functional indicator within the present dataset.

PCA based on the four radical scavenging activities further illustrated the heterogeneity of antioxidant responses (**Figure 5C**). The first two principal components explained 67.9% of the total variance, with PC1 and PC2 accounting for 40.1% and 27.8%, respectively. Samples from different production years showed partial separation, although some years overlapped. The loading vectors indicated that ·OH and PTIO· contributed in opposite directions, whereas DPPH· and ${{\text{O}}_{2}}^{-}\cdot$ were more closely grouped. This pattern suggests that different antioxidant assays capture distinct functional dimensions of the tea infusion. Pearson correlation analysis was then performed to examine the relationships between antioxidant activities and selected chemical and sensory attributes using year means (n = 8) (**Figure 5D**). PTIO· scavenging showed the strongest positive correlation with total polyphenols (r = +0.84, *p* < 0.01) and was also positively associated with several fresh-tea-related sensory attributes, including Brisk, Sweet, Umami, and liquor color. In contrast, it was negatively associated with aged-tea-related attributes such as Sour and Smooth, as well as with storage-associated chemical variables such as caffeine and theabrownins. These correlations support the interpretation that PTIO· scavenging reflects a functional dimension more closely related to fresh-tea chemical and sensory characteristics. In contrast, ·OH scavenging showed an opposite tendency, being positively associated with storage-related attributes such as Sour and Smooth and negatively associated with fresh-tea attributes such as Brisk and Umami. DPPH· scavenging showed moderate but non-significant correlations with polyphenol-related variables, whereas ${{\text{O}}_{2}}^{-}\cdot$ scavenging showed weak associations with most chemical and sensory attributes, consistent with its limited variation among storage years. Together, these results indicate that different antioxidant assays reflect distinct antioxidant pools and respond differently to storage-associated compositional changes.

Overall, antioxidant profiling revealed that long-term storage affects the functional properties of Lapsang Souchong in a radical-specific manner. PTIO· scavenging showed a significant storage-related decline and was closely associated with fresh-tea chemical and sensory characteristics, whereas ·OH scavenging displayed a contrasting response pattern. These findings suggest that antioxidant activity during storage is not governed by a single overall increase or decrease, but by the differential transformation of multiple antioxidant-related compound pools.

### 3.6 Multi-modal integration and quantitative framework for storage-related quality evaluation

After characterizing storage-related changes at the sensory, chemical, metabolomic, and functional levels, we further integrated these datasets to evaluate whether the storage trajectory of Lapsang Souchong could be summarized by a quantitative multi-modal framework. Nineteen key variables were selected from the preceding analyses, representing five analytical modalities: sensory attributes, electronic tongue responses, chemical components, storage-associated metabolites, and antioxidant activities. These variables included Overall score, Liquor color, Sour, Smooth, Brisk, electronic tongue Sourness, Bitterness, and Astringency, Polyphenols, Theaflavins, Thearubigins, Theabrownins, LysoPC(19:2), Abietic acid, Dehydro-EGC-Glc-Ara, Hydroxyricinoleic acid, PTIO· scavenging, ·OH scavenging, and DPPH· scavenging. The raw data matrix used for correlation analysis and PLSR modeling is provided in **Supplementary Table 2**.

**Figure 6:**
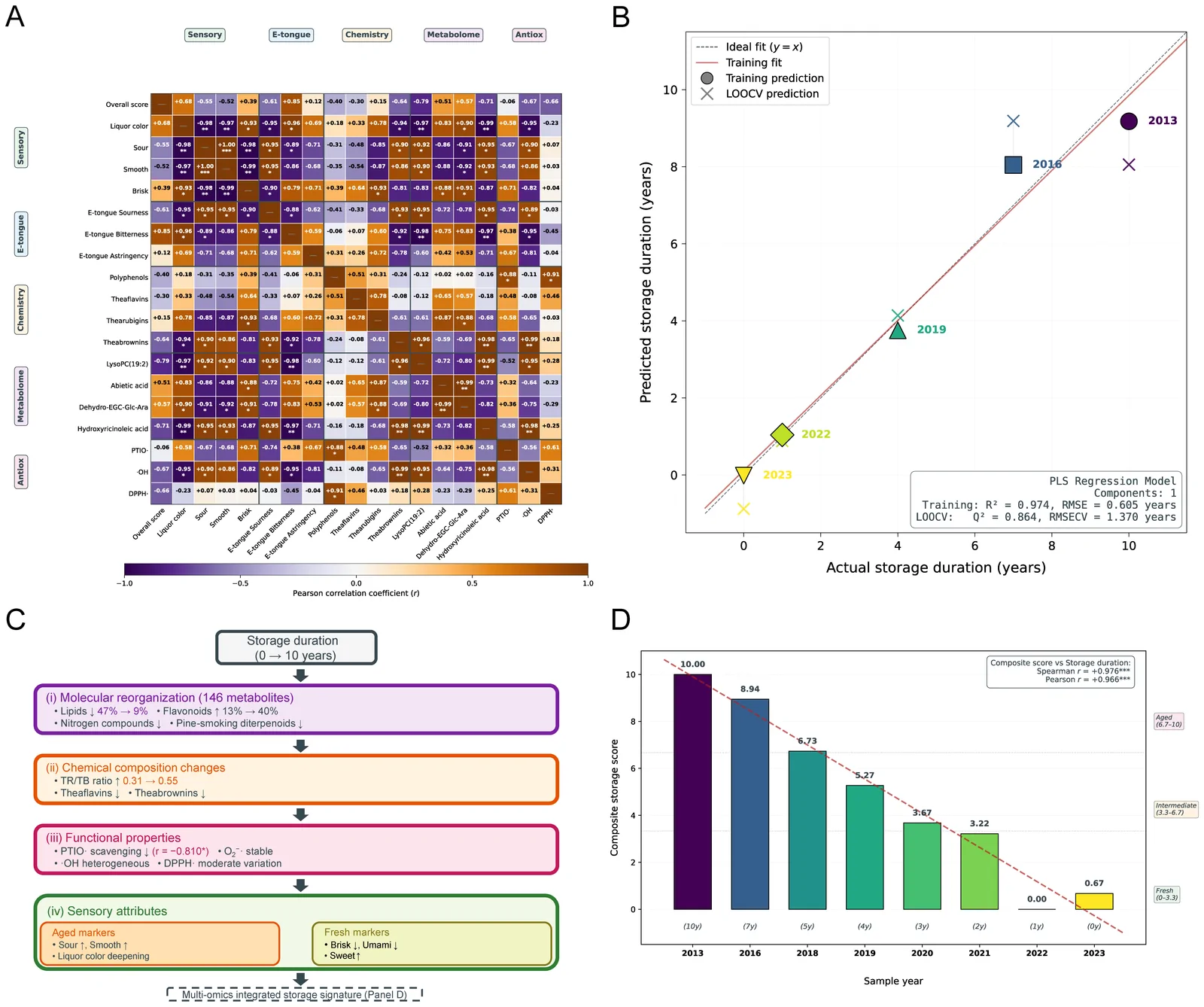
Multi-modal integration of storage-associated quality changes in Lapsang Souchong black tea. **(A)** Pearson correlation matrix of 19 key variables representing five analytical modalities: sensory attributes, electronic tongue responses, chemical components, storage-associated metabolites, and antioxidant activities. Correlations were calculated using year means from five representative production years (2013, 2016, 2019, 2022, and 2023). Color intensity represents Pearson correlation coefficients, and significance levels are indicated by asterisks. **(B)** Partial least squares regression (PLSR) model for predicting storage duration based on the 19 variables. Filled symbols indicate training predictions, the cross (×) indicate leave-one-out cross-validation (LOOCV) predictions, the dashed line indicates the ideal fit (y = x), and the solid line indicates the training regression fit. The optimal one-latent-variable model showed good performance, with training *R^2^* = 0.974, RMSE = 0.605 years, LOOCV *Q^2^* = 0.864, and RMSECV = 1.370 years. **(C)** Conceptual summary of multi-level storage-associated changes in Lapsang Souchong black tea. Long-term storage was associated with coordinated changes in metabolite composition, tea pigment chemistry, antioxidant activity, and sensory attributes, collectively reflecting the transition from fresh-tea characteristics to aged-tea characteristics. **(D)** Composite storage score across eight production years. The score was calculated as a weighted combination of seven Z-score-standardized indicators: Sour, Theabrownins, Smooth, Brisk, PTIO· scavenging, Liquor color, and Theaflavins. Raw scores were normalized to a 0–10 scale, and horizontal lines indicate the Fresh, Intermediate, and Aged stages. The composite score showed strong concordance with actual storage duration (Spearman r = +0.976, *p* < 0.001; Pearson r = +0.966, *p* < 0.001).

Pearson correlation analysis of the 19 selected variables was performed using year means from the five samples with complete metabolomic data: 2013, 2016, 2019, 2022, and 2023 (**Figure 6A**). Strong correlations were observed not only within individual modalities but also across modalities, including sensory–chemical, sensory–metabolite, chemical–antioxidant, and metabolite–antioxidant associations. These cross-modal correlations indicated that the storage-related changes described in the preceding sections were not isolated responses, but were interconnected across sensory perception, chemical composition, metabolite remodeling, and antioxidant activity.

To further evaluate whether the integrated variable set could predict storage duration, partial least squares regression (PLSR) was performed using the 19 variables as predictors and storage duration as the response. The optimal number of latent variables was selected by leave-one-out cross-validation (LOOCV). A single-latent-variable model showed the best cross-validation performance, with *Q^2^* = 0.864 and RMSECV = 1.370 years, whereas adding additional latent variables did not improve model performance (**Supplementary Table 3**). The final model showed good fitting performance, with training *R^2^* = 0.974 and RMSE = 0.605 years (**Figure 6B**). Although the model was based on a limited number of samples, the results suggest that the integrated quality profile captured the major storage-related variation in Lapsang Souchong. Given the limited number of production years with complete multimodal data, this PLSR model should be regarded as an exploratory proof-of-concept tool for summarizing storage-associated variation within the present dataset rather than as a finalized model for practical storage-age authentication. The sample-level training predictions, LOOCV predictions, and corresponding residuals are provided in **Supplementary Table 5**.

Variable importance in projection (VIP) analysis was used to identify the major contributors to the PLSR model (**Supplementary Table 4**). Twelve of the 19 variables showed VIP values greater than 1.0. These variables were distributed across all five modalities, including sensory attributes (Sour, Liquor color, Smooth, and Brisk), electronic tongue responses (Sourness and Bitterness), storage-associated metabolites (Hydroxyricinoleic acid, LysoPC(19:2), Dehydro-EGC-Glc-Ara, and Abietic acid), one chemical component (Theabrownins), and one antioxidant activity indicator (·OH scavenging). The highest VIP values were observed for Sour, Liquor color, electronic tongue Sourness, and Smooth, indicating that sensory and instrumental taste variables were among the most sensitive indicators of storage duration. The inclusion of lipid-related metabolites and pine-smoking-associated diterpenoids among the important variables further suggests that both general storage reactions and Lapsang Souchong-specific chemical features contributed to the integrated storage signature.

A composite storage score was further calculated using seven core storage-associated indicators selected from the univariate and integrative analyses, including Sour, Theabrownins, Smooth, Brisk, PTIO· scavenging, Liquor color, and Theaflavins. The detailed weighting scheme is provided in **Supplementary Table 6**. After normalization to a 0–10 scale, the composite score showed a clear storage-related trend across the eight production years (**Figure 6D**), with higher scores in long-stored samples and lower scores in fresh samples. The normalized scores were 10.00, 8.94, 6.73, 5.27, 3.67, 3.22, 0.00, and 0.67 for the 2013, 2016, 2018, 2019, 2020, 2021, 2022, and 2023 samples, respectively. The complete Z-score calculation is provided in **Supplementary Table 7**. Concordance analysis showed strong agreement between the composite score and actual storage duration (Spearman r = +0.976, *p* < 0.001; Pearson r = +0.966, *p* < 0.001; **Supplementary Table 8**). The slight inversion between the 2022 and 2023 samples was mainly associated with the relatively high PTIO· scavenging activity of the 2022 sample. The composite score was intended as a simplified descriptive index rather than a standardized grading model. Therefore, the Fresh, Intermediate, and Aged categories were used only to facilitate visualization and interpretation within the present dataset.

Finally, a conceptual model was constructed to summarize the multi-level storage-associated changes in Lapsang Souchong (**Figure 6C**). Long-term storage was associated with metabolite remodeling, including changes in lipid-derived compounds, flavonoid- and tannin-related metabolites, and pine-smoking-associated diterpenoids. These molecular changes were accompanied by shifts in tea pigment chemistry, especially changes in Theabrownins, Theaflavins, and the TR/TB ratio, as well as radical-specific antioxidant responses. At the sensory level, these changes were reflected by increased Sour and Smooth attributes and decreased Brisk, Umami, and Sweet attributes. Together, the correlation matrix, PLSR model, VIP analysis, and composite score indicate that storage-related quality transformation in Lapsang Souchong involves coordinated changes across multiple analytical levels rather than a simple uniform decline in quality.

## 4 Discussion

Long-term storage of black tea is often associated with the loss of fresh-tea characteristics and the development of aged-tea attributes (Ho et al., 2015; Xu et al., 2021). In this study, a decade-long, multi-modal investigation of Lapsang Souchong black tea showed that storage did not simply cause uniform quality deterioration, but reshaped tea quality through coordinated changes in sensory perception, taste-related chemistry, metabolite composition, and antioxidant activity. This storage-related quality trajectory was characterized by increased Sour and Smooth attributes, decreased Brisk, Umami, and Sweet attributes, pigment-related chemical transformation, metabolite-class remodeling, and radical-specific antioxidant responses. These findings provide the basis for discussing the multi-level coordination of Lapsang Souchong storage transformation, the functional significance of PTIO· scavenging, the storage-related depletion of pine-smoking-derived diterpenoids, the agreement between QDA and electronic tongue profiling, and the applicability and limitations of the proposed quantitative framework for storage-related quality evaluation.

### 4.1 The coordinated multi-level nature of Lapsang Souchong storage transformation

The integrated analysis suggested that storage-related changes in Lapsang Souchong occurred across multiple analytical levels rather than within a single sensory or chemical dimension. Pearson correlation analysis of 19 representative variables showed extensive associations among sensory attributes, electronic tongue responses, chemical components, storage-associated metabolites, and antioxidant activities (**Figure 6A**). Although the number of samples with complete metabolomic data was limited, the presence of strong cross-modal correlations indicates that the observed storage-related changes were interconnected across sensory, chemical, molecular, and functional dimensions. This coordinated pattern can be interpreted through the conceptual framework summarized in **Figure 6C**.

At the metabolite level, widely targeted metabolomics identified 183 storage-associated metabolites, revealing marked changes in several compound classes, including lipid-derived metabolites, flavonoid- and tannin-related compounds, terpenoids, lignans and phenolic compounds. The accumulation of lipid-derived compounds such as LysoPC(19:2) and hydroxyricinoleic acid suggests that lipid hydrolysis and oxidation are important processes during long-term storage. Meanwhile, the depletion of flavonoid glycosides, tannin-related compounds, phenolic acid derivatives, and pine-resin-related diterpenoids indicates progressive transformation or loss of storage-sensitive molecular constituents. In particular, the marked decreases in abietic acid, dehydroabietic acid, and 12-hydroxymethylabietate suggest that resin-associated diterpenoids derived from or related to pinewood smoking may gradually diminish during long-term storage. These metabolite-level changes were accompanied by shifts in pigment-related chemistry. In particular, theabrownins were higher in long-stored samples, whereas thearubigins were relatively higher in fresher samples. Consequently, the TR/TB ratio was lower in aged samples and higher in fresh samples, indicating that a reduced TR/TB ratio may reflect a more advanced storage-related chemical state. This pattern is consistent with the progressive oxidation, condensation, and polymerization of phenolic compounds during black tea storage, in which lower-molecular-weight tea pigments and polyphenolic precursors are gradually transformed into more polymerized brown pigments. The chemical and metabolite changes were further linked to functional and sensory responses. Antioxidant assays showed radical-specific storage responses rather than a uniform increase or decrease in antioxidant activity. PTIO· scavenging declined with storage duration and was associated with fresh-tea-related attributes, whereas ·OH scavenging showed a contrasting response pattern more closely aligned with aged-tea characteristics. At the sensory level, these functional and chemical changes were reflected by the accumulation of Sour and Smooth attributes and the decline of Brisk, Umami, and Sweet attributes. Thus, the storage transformation of Lapsang Souchong can be understood as a coordinated process involving molecular remodeling, pigment conversion, antioxidant response changes, and sensory reorganization.

This interpretation differs from a simple degradation model of tea storage. Rather than indicating indiscriminate loss of quality, the results suggest that long-term storage reshapes the quality profile of Lapsang Souchong in an attribute-specific and chemically structured manner. Similar coordinated aging phenomena have been reported in other tea types, including white tea, oolong tea, Qingzhuan tea, and Pu-erh tea (Cheng et al., 2021; Hong et al., 2021; Chen et al., 2025; Li et al., 2026). The present study extends this concept to Lapsang Souchong by integrating sensory evaluation, electronic tongue analysis, chemical composition, metabolomics, antioxidant activity, PLSR modeling, and a composite storage score. Together, these results support the view that Lapsang Souchong storage represents an ordered multi-level quality transformation rather than random quality deterioration.

### 4.2 PTIO· scavenging activity as a storage-responsive functional indicator

Among the four antioxidant assays examined in this study, PTIO· scavenging showed the clearest monotonic association with storage duration (Spearman r = −0.810, *p* = 0.015; **Figure 5B**). This pattern differed from the more irregular changes observed for DPPH·, ·OH, and ${{\text{O}}_{2}}^{-}\cdot$ scavenging, suggesting that PTIO· scavenging may represent a storage-responsive functional indicator in Lapsang Souchong black tea. PTIO· is a stable oxygen-centered radical that can be used as a hydrophilic ROS-mimicking radical in aqueous antioxidant assays (Li et al., 2018; Matemu et al., 2021). In the present study, the storage-related decrease in PTIO· scavenging may reflect the depletion or transformation of water-soluble antioxidant constituents during long-term storage. This possibility is supported by the metabolomic results, which showed that nitrogen-containing metabolites and amino acid derivatives tended to decrease in long-stored samples (**Figure 4**). Given that amino acid derivatives and other nitrogen-containing compounds may contribute to antioxidant activity through hydrogen donation, electron transfer, and radical-scavenging mechanisms (Matemu et al., 2021), their depletion could partly explain the reduced PTIO· scavenging capacity. However, this interpretation remains hypothesis-generating and requires further experimental validation using targeted quantification and mechanistic antioxidant assays. Therefore, PTIO· scavenging should be regarded as a storage-responsive functional indicator within the present dataset rather than as a confirmed mechanistic marker.

The correlation patterns further supported the distinct behavior of PTIO· scavenging. PTIO· scavenging was positively associated with fresh-tea-related attributes and components, including Brisk, Sweet, Umami, and total polyphenols, but negatively associated with aged-tea-related attributes such as Sour and Smooth, as well as with storage-associated chemical variables such as caffeine and theabrownins (**Figure 5D**). In contrast, ·OH scavenging showed an opposite tendency, being more closely associated with aged-tea characteristics. The opposing loading directions of PTIO· and ·OH scavenging in the PCA biplot also suggested that these two assays reflected different antioxidant response dimensions during storage (**Figure 5C**). Thus, PTIO· and ·OH scavenging may provide complementary information for understanding functional changes in stored black tea. Notably, although PTIO· scavenging showed a significant univariate correlation with storage duration, its VIP value in the PLSR model was moderate and below the conventional threshold of 1.0 (VIP = 0.801; **Supplementary Table 4**). This result is not contradictory. In a multivariate model, VIP values reflect the unique contribution of each variable after accounting for correlations with other predictors. The moderate VIP value of PTIO· scavenging suggests that part of its storage-related information overlapped with other variables, especially sensory attributes and pigment-related chemical indicators. Therefore, the value of PTIO· scavenging lies not primarily in being the strongest independent predictor in the integrated model, but in providing a simple and functionally interpretable assay that reflects one aspect of storage-related biochemical transformation.

From a practical perspective, PTIO· scavenging may serve as a useful complementary indicator for evaluating storage-related functional changes in Lapsang Souchong. Compared with metabolomic profiling, the PTIO· assay is simpler and more accessible, making it potentially suitable for rapid screening or quality monitoring. However, its broader applicability requires further validation using independent sample batches, different storage conditions, and other black tea types. Therefore, PTIO· scavenging may function as a potential functional chronometer of storage progression in Lapsang Souchong, although further validation is required.

### 4.3 Pine-smoking-derived diterpenoids as Lapsang Souchong-specific storage-associated markers

A distinctive finding of the metabolomic analysis was the storage-related depletion of pine-smoking-derived diterpenoids, particularly abietic acid and dehydroabietic acid. These compounds were much more abundant in the fresh 2023 sample than in the 10-year-stored 2013 sample, with approximately 18- and 13-fold differences, respectively (**Figure 4**; **Supplementary Table 1**). This pattern is closely related to the traditional processing of Lapsang Souchong, in which tea leaves are smoked over pine wood during withering and drying, allowing pine-derived compounds to be deposited on or absorbed into the tea matrix (Yao et al., 2005). Abietic acid and dehydroabietic acid are resin-derived diterpenoid acids associated with conifer wood and pine smoke. Abietic acid is a major resin acid in pine oleoresin, whereas dehydroabietic acid is an oxidized or aromatized derivative formed during thermal processing and environmental transformation (Simoneit et al., 2000; Simoneit, 2002). Their high abundance in fresh Lapsang Souchong therefore provides a non-volatile chemical signature of the pinewood-smoking process, complementing previously reported volatile smoke-related compounds such as longifolene, longicyclene, guaiacol, 4-methylguaiacol, and 4-ethylguaiacol (Yao et al., 2005).

The marked decrease in abietic acid and dehydroabietic acid during storage may be associated with oxidative transformation of resin-acid structures, gradual loss or desorption of smoke-derived constituents, and further reactions or interactions with tea matrix components. These two compounds were annotated based on the widely targeted metabolomic database by matching precursor ions, product ions, and retention times. Although they are closely related to conifer resin and pinewood smoke, their identities and storage-related changes should be further confirmed using authentic standards, targeted quantitative analysis, and independent sample sets. Therefore, in the present study, abietic acid and dehydroabietic acid should be regarded as candidate process-related molecular indicators rather than definitive authenticity or aging markers of Lapsang Souchong black tea. Abietic acid and dehydroabietic acid are characteristic diterpenoid resin acids derived from conifer resin, and dehydroabietic acid has been widely used as an organic tracer of conifer smoke. Given that pinewood smoking is a defining processing step of traditional Lapsang Souchong, these compounds likely represent non-volatile chemical signatures of pinewood smoke deposition during processing (Rogge et al., 1998; Simoneit, 2002; Yao et al., 2005). In addition, Pinus resin acids are known to undergo ageing-related molecular changes, including oxidative conversion of abietane-type resin acids into dehydroabietic acid and more oxidized derivatives (Beltran Sanchidrian et al., 2016). Although the exact transformation pathway of these diterpenoids in the tea matrix requires further verification, their strong storage-related decline suggests that pine-derived diterpenoids may serve as characteristic process-related molecular indicators of Lapsang Souchong aging. Unlike general storage indicators such as Sour, Smooth, theabrownins, or PTIO· scavenging, these diterpenoids are closely linked to the unique smoking process of Lapsang Souchong and may therefore provide variety- and process-specific information. In the PLSR model, abietic acid contributed to storage-duration prediction with a VIP value slightly above the conventional threshold of 1.0 (VIP = 1.013; **Supplementary Table 4**). However, its VIP value was lower than those of the leading sensory and electronic tongue variables, including Sour, liquor color, electronic tongue Sourness, and Smooth. This indicates that abietic acid was not the dominant general predictor of storage duration, but rather a Lapsang Souchong-specific chemical feature that complemented the broader sensory and chemical indicators. In this sense, pine-derived diterpenoids may help capture the specialty-specific dimension of Lapsang Souchong storage transformation.

From an application perspective, these compounds may have potential value in authenticity evaluation and storage-related quality assessment of Lapsang Souchong. Their presence at relatively high abundance may help distinguish pine-smoked Lapsang Souchong from non-smoked black teas, while their gradual depletion may contribute to the mellowing of the characteristic smoky profile during storage. However, this interpretation should be further validated using independent sample batches, controlled storage experiments, volatile profiling, and comparisons with other smoked and non-smoked black teas. Future studies integrating volatile and non-volatile metabolomics will be particularly useful for clarifying how pinewood-smoking-derived compounds shape the aroma evolution of stored Lapsang Souchong.

### 4.4 Methodological and practical implications of the integrated evaluation framework

The agreement between QDA and electronic tongue profiling provides methodological support for the sensory findings of this study. Both approaches captured the major storage-related taste shift, especially the increase in sourness and the transition from fresh-tea attributes toward aged-tea attributes. Among the matched sensory–instrumental pairs, electronic tongue Sourness showed the strongest correspondence with QDA Sour, indicating that sourness was the most robust cross-modal taste indicator. However, the weaker correspondence observed for bitterness, astringency, umami, and sweetness also indicates that electronic tongue responses cannot fully replace expert sensory evaluation, particularly for complex mouthfeel and integrated quality perception. Therefore, electronic tongue profiling is best regarded as a complementary tool for rapid and standardized quality screening. The PLSR model and composite storage score further translated the multi-modal findings into a quantitative framework for storage-related quality evaluation. The single-latent-variable PLSR model captured the major storage-associated variation, and VIP analysis showed that important predictors were distributed across sensory, electronic tongue, chemical, metabolomic, and antioxidant modalities. This distributed contribution supports the view that no single indicator fully represents Lapsang Souchong storage transformation. In parallel, the composite storage score provided a simpler and more interpretable index based on seven core indicators, including Sour, Theabrownins, Smooth, Brisk, PTIO· scavenging, Liquor color, and Theaflavins. Compared with the full PLSR model, this score may be more practical for routine quality evaluation because it uses fewer variables and can be calculated without complex multivariate modeling. From an application perspective, with further validation, the proposed framework may provide useful reference points for future storage-related quality evaluation, quality monitoring, and authenticity assessment of Lapsang Souchong. Sensory attributes and electronic tongue responses provide rapid quality information, tea pigment variables reflect chemical aging, pine-smoking-derived diterpenoids offer process-specific information, and antioxidant assays provide functional indicators. Nevertheless, these applications should be considered preliminary until the framework is validated using independent sample sets and controlled storage experiments.

### 4.5 Limitations and future perspectives

Several points should be considered when interpreting these findings. First, the present study used naturally stored Lapsang Souchong samples from different production years rather than a controlled same-batch aging experiment. Therefore, storage duration was inherently confounded with production year, and part of the observed variation may reflect annual differences in raw material composition, processing background, batch characteristics, or storage microenvironment in addition to storage duration itself. Accordingly, the identified changes should be interpreted as storage-associated patterns within the present natural storage-year series rather than as definitive causal effects of storage duration alone. Although all samples were stored under comparable warehouse conditions, continuous records of temperature, relative humidity, oxygen exposure, and light exposure during the entire storage period were not available, which may also have contributed to part of the year-to-year variation observed in this study. Future controlled storage experiments using the same initial tea batch will therefore be necessary to validate the causal contribution of storage duration to these quality changes. Second, although the single-latent-variable PLSR model showed good cross-validation performance in the present dataset, it should be interpreted cautiously because it was constructed using only five representative production years with complete multimodal data. The model provides an exploratory framework for integrating multiple storage-associated variables, but its generalizability and practical applicability require validation using larger independent sample sets and controlled storage experiments. Third, because Lapsang Souchong has a unique pinewood-smoking process, the pine-derived diterpenoid markers identified in this study may be specific to this tea type. Future studies should validate these candidate indicators using authentic standards, targeted quantification, independent natural storage sample sets, controlled same-batch storage experiments, and comparative analyses among smoked Lapsang Souchong, non-smoked Lapsang Souchong, and other black teas. Fourth, the storage-associated metabolites were identified using a two-step screening strategy that combined extreme-group comparison with correlation analysis within the same dataset. Although this approach helped prioritize metabolites showing both large differences between fresh and aged samples and monotonic storage-associated trends, it may introduce selection bias and should therefore be regarded as a candidate-discovery strategy. Independent sample sets and targeted validation are needed to confirm these metabolites as robust storage-related indicators. Finally, the weighting scheme of the composite storage score was designed to be interpretable, but it was not optimized using an external dataset. Future studies should compare this expert-selected weighting strategy with alternative approaches, such as equal-weight scoring, PLSR coefficient-based weighting, machine learning-derived weighting, and external validation-based optimization.

Overall, this study shows that long-term storage of Lapsang Souchong black tea involves coordinated changes in sensory attributes, tea pigment chemistry, metabolite composition, and antioxidant activity. The storage trajectory was characterized by increased Sour and Smooth attributes, decreased fresh-tea taste characteristics, accumulation of theabrownins, depletion of selected pine-smoking-derived diterpenoids, and radical-specific antioxidant responses. The integrated PLSR model and composite storage score further indicate that storage-related quality transformation can be quantitatively summarized using a limited set of multi-modal indicators. These findings provide a systematic basis for understanding the biochemical and sensory evolution of stored Lapsang Souchong and offer preliminary reference points for future quality evaluation, storage management, and authenticity assessment, and pending validation with broader sample sets and controlled storage experiments.

## Data Availability

The original contributions presented in the study are included in the article/**Supplementary Material**. Further inquiries can be directed to the corresponding author.
