# Supplementary material for: Integrated sensory, chemical, metabolomic, and antioxidant profiling reveals coordinated quality transformation of Lapsang Souchong black tea during decade-long storage
Source: Front Plant Sci. 2026 Aug 20;17:1934917. doi: 10.3389/fpls.2026.1934917 (PMC13538424; doi:10.3389/fpls.2026.1934917)
Supplement: Supplementary file 1 [file Image1.pdf]

Supplementary Material

Supplementary Figures

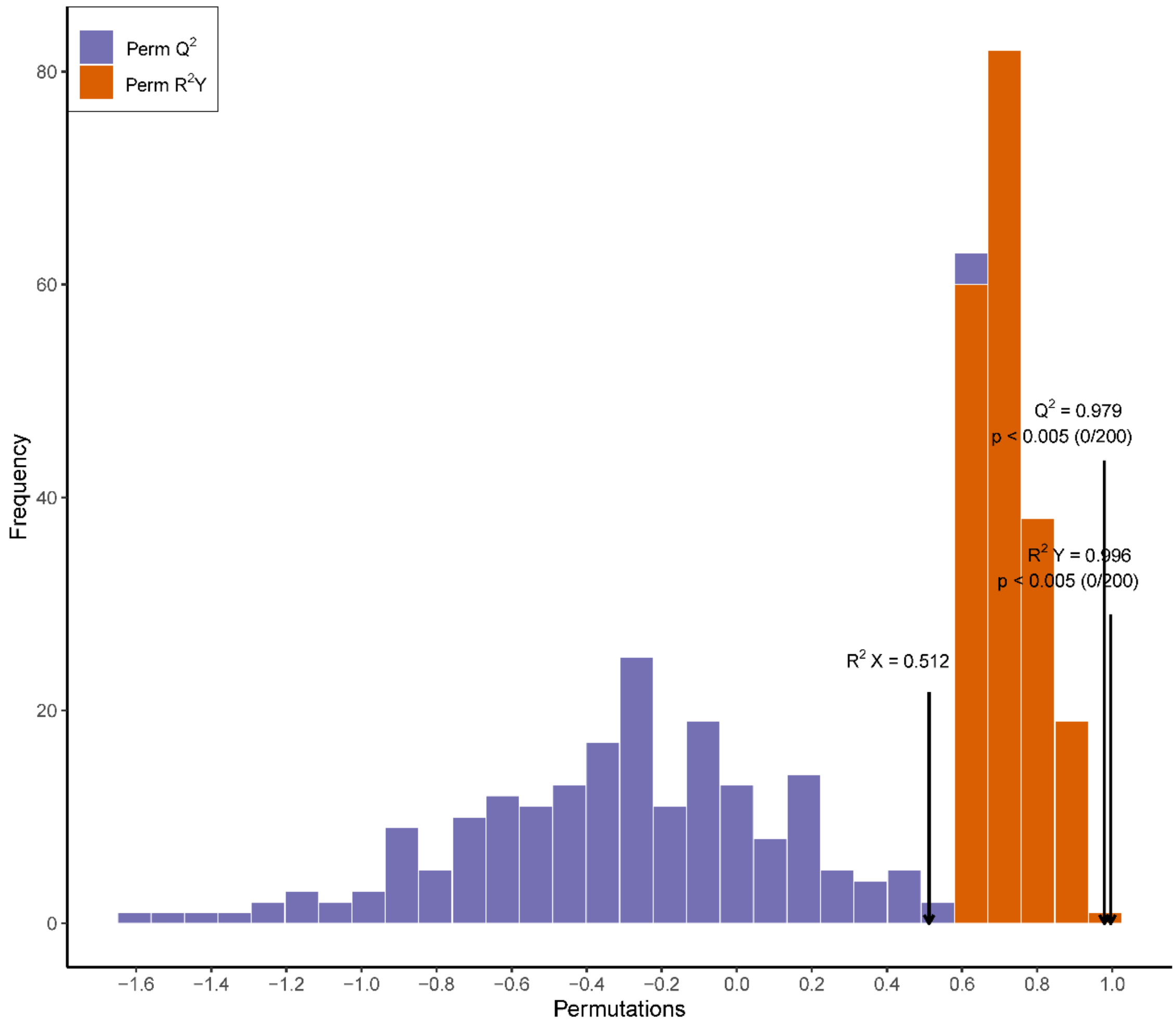

Supplementary Figure S1. Validation of the OPLS-DA model by 200 permutation tests. The histogram shows the distributions of permuted Q^2 and R^2Y values generated from 200 random permutations. Arrows indicate the corresponding values of the original OPLS-DA model, including $R^2X = 0.512$, $R^2Y = 0.996$, and $Q^2 = 0.979$. The permutation test showed that the original model had significantly higher R^2Y and Q^2 values than the permuted models, supporting the robustness of the OPLS-DA model.
